# Identification and construction of a novel biomimetic delivery system of paclitaxel and its targeting therapy for cancer

**DOI:** 10.1038/s41392-020-00390-6

**Published:** 2021-01-27

**Authors:** Xue Wang, Wanwei Zheng, Qing Shen, Yahua Wang, Yujen Tseng, Zhongguang Luo, Xiaoyou Wang, Lei Shi, Chong Li, Jie Liu

**Affiliations:** 1 Medical Research Institute, College of Pharmaceutical Sciences, Southwest University, Chongqing, 400715 China; 2 Institute of Digestive Diseases, Huashan Hospital, Fudan University, Shanghai, 200041 China; 3 Hangzhou YITU Healthcare Technology Co., Ltd, Hangzhou, 310012 China; 4 State Key Laboratory of Esophageal Cancer Prevention & Treatment, Zhengzhou University, Zhengzhou, 450052 China; 5 Xiamen Ginposome Pharmaceutical Co., Ltd, Xiamen, 361026 China

**Keywords:** Drug development, Drug delivery

**Dear Editor**,

Paclitaxel (PTX) is among the first-line therapeutic agents against diverse malignancies. However, problems still exist concerning its poor water-solubility, multidrug resistance, lack of specific targeting ability and severe toxic effects, and these issues are far from fully resolved despite the various PTX formulations available on the market, e.g. the gold standard, PTX albumin nanoparticles Abraxane^®^,^1^ and PTX liposomes Lipusu^®^. Some studies that try to solve the multiple problems faced by chemotherapy drug delivery, however, fell into the prevailing trap of overcomplicated formulation design which sacrifices the druggability.^2^ To better reconcile this paradox, a novel glycosylated liposomal PTX was designed, inspired by the cytomembrane glycosyls with important roles in maintaining both membrane structure and physiological functions,^3,4^ such as enhancing membrane stability, evading immunological clearance, and recognizing corresponding receptors. Ginsenosides, as natural amphiphilic molecules, are structurally similar to cholesterol, contain glycosyl moieties, and are easily obtainable. Thus, we adopted ginsenoside as a substitute for cholesterol in liposomes, with its hydrophobic region inserted into the lipid bilayer, and its glycosyl exposed on the liposomal surface providing biomimetic functions. This ginsenoside-anchored liposome was named ginposome, and the paclitaxel-loaded ginposome (G-PTX) system exhibited enhanced encapsulation and stability, reduced monocyte phagocytosis, and active targeting towards tumor cells by recognizing their overexpressed glucose transporters. In addition, the ginsenoside could also serve as chemotherapy adjuvant due to its inherent pharmacological functions.^5^

First, we screened for the most desirable ginsenoside as the cholesterol substitute. Ginsenoside Rg5, with a disaccharide group at the 3-position of the skeleton, showed effective stabilization of the liposome membrane (Fig. 1a, b), and this was consistent with the molecular dynamic calculation results which confirmed that the glycosyl moiety at the 3-position oriented towards the water molecules without disruption of phospholipid arrangement (Supplementary Fig. S1a, b). Meanwhile, the binding affinity between Rg5 and GLUT1 experienced no interference at high concentration of free glucose (Fig. 1c and Supplementary Fig. S2a, b), which was further verified by the cellular internalization assay under high-glucose condition (Supplementary Fig. S2c–e). Therefore, ginsenoside Rg5 was adopted as a cholesterol substitute for the construction of novel PTX liposomes.

**Fig. 1:**
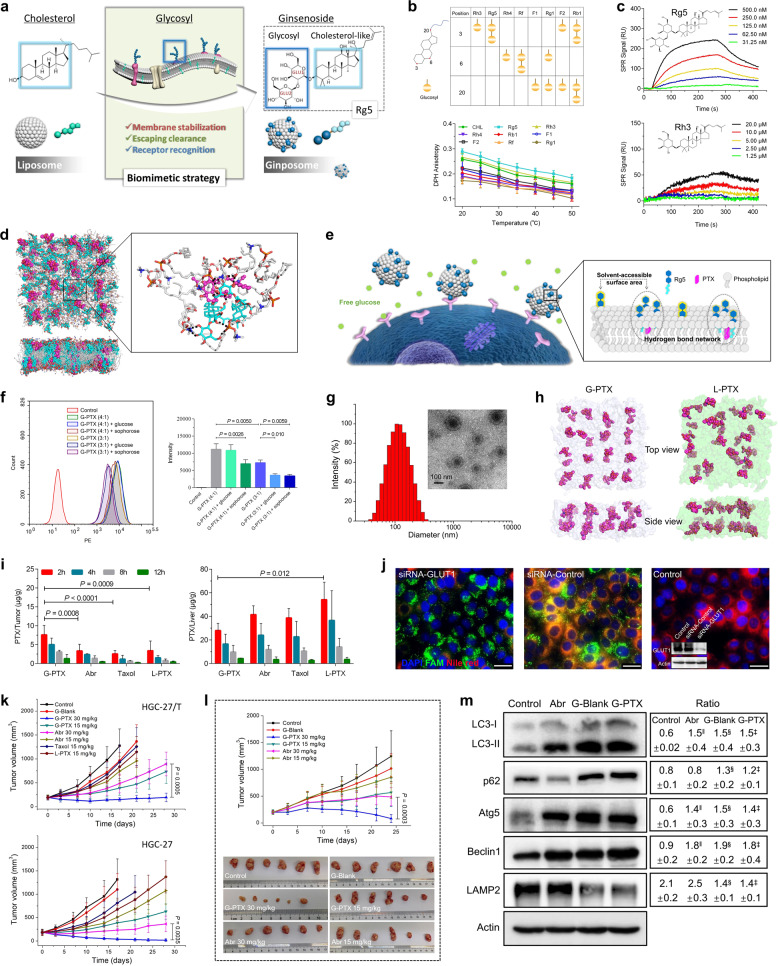
Paclitaxel-loaded ginsenoside-anchored liposome (Ginposome-PTX) with the simple formulation and advanced functions. **a** Schematic view of the ginsenoside-anchored liposome (ginposome) design. **b** The eight representative natural ginsenosides for screening, and their effects on diphenylhexatriene (DPH) anisotropy in liposomes. These ginsenosides share similar skeletons, and all their glycosyls consisted of completely glucose units, yet with different numbers and carbon positions. Conventional cholesterol-containing liposome (CHL) was adopted as control (*n* = 3). **c** The influence of free glucose (25 mM) on the interaction between GLUT1 and Rg5 or Rh3 by surface plasma resonance (SPR) analysis. **d** Snapshot of the lipid bilayer of G-PTX, and the hydrogen-bond networks formed around PTX. POPC (white) and Rg5 (blue) are shown by stick, hydrogen-bonds are denoted by black dashed lines, and PTX (purple) is shown by the sphere. **e**, **f** Surface glycosyl with sufficient SASA was required for G-PTX to realize its active-targeting ability, shown by a schematic diagram and quantitative analysis by flow cytometry (*n* = 3; one-way ANOVA). **g** Size distribution and TEM image (inlet) of G-PTX. **h** Snapshots of G-PTX and L-PTX (conventional liposomal PTX) after 200 ns molecular dynamics simulations, respectively. Paclitaxel molecules, conventional liposome bilayer, and Rg5-anchored lipid bilayer are colored in purple, green, and light red, respectively. **i** In vivo distribution of different paclitaxel formulations in tumor and liver of HGC-27 xenograft mice models (*n* = 3; two-way ANOVA). **j** Direct inhibition of GLUT1 transporter via siRNA transfection reduced G-PTX uptake in HGC-27 cancer cells, by fluorescence microscopy and western blot (inlet). siRNA-GLUT1 or a scrambled siRNA (siRNA-Control) was modified by FAM (green) and G-PTX was labeled by Nile red. Scale bar, 25 μm. **k** Tumor growth curves in different groups on HGC-27/T and HGC-27 tumor models (*n* = 6; two-tailed *t*-test). **I** The in vivo antitumor effects of G-PTX and Abraxane on PDX models. The tumors at the end of the experiment were photographed (*n* = 6; two-tailed *t*-test). **m** The expression of autophagy-related proteins in the HCC-27/T cells treated by G-PTX, G-Blank or Abraxane (*n* = 3; one-way ANOVA). Data were expressed as mean ± s.d. ^‖^*P* < 0.05 versus Control, ^§^*P* < 0.05 versus Control, ^‡^*P* < 0.05 versus Control. Exact *P* values: Abraxane versus Control, 0.046 (LC3-II), 0.0305 (Atg5), 0.0159 (Beclin1); G-Blank versus Control, 0.0316 (LC3-II), 0.0119 (p62), 0.023 (Atg5), 0.0093 (Beclin1), 0.0152 (LAMP2); G-PTX versus Control, 0.0385 (LC3-II), 0.0213 (p62), 0.0351 (Atg5), 0.0116 (Beclin1), 0.023 (LAMP2)

The formulation of G-PTX was then optimized according to the stability and in vitro targeting ability. Molecular dynamics simulations showed that Rg5 formed intensive hydrogen bonds with POPC and PTX, which further constituted hydrogen bond networks to stabilize the entire system (Fig. 1d and Supplementary Fig. S3a). With the increase of the Rg5/PTX molar ratio, the stability of G-PTX raised and reached a plateau at 3:1 Rg5/PTX ratio, surpassing the stability of conventional paclitaxel liposome (L-PTX) containing an identical proportion of cholesterol (Supplementary Fig. S3b, c). High-glucose level reduced the cellular uptake of G-PTX at 3:1 Rg5/PTX ratio, possibly related to the decreased solvent-accessible surface area (SASA) of the disaccharide in Rg5 along with the formation of hydrogen bond networks (Fig. 1e, f). When the Rg5/PTX ratio reached 4:1, its in vitro targeting function peaked, and further increases of the ratio engendered no more improvement (Supplementary Fig. S3d). To facilitate the quantitative preparation of G-PTX, a 4.5:1 molar ratio (equivalent to 4:1 mass ratio) was selected. The final G-PTX had a spherical shape with an average particle size of ~110 nm, encapsulation efficiency of 97.2% and maintained high stability even when the PTX concentration reached over 5 mg/mL (Fig. 1g and Supplementary Fig. S3e–j). Molecular dynamics simulation also confirmed the marked improvement in integral stability of G-PTX (Fig. 1h and Supplementary Fig. S4a–d).

We further investigated its expected long circulation and active-targeting function in vivo. The elevated surface hydrophilicity of G-PTX provided by glycosyls partly reduced the formation of protein corona thus decreased the macrophage uptake (Supplementary Fig. S5a, b). Pharmacokinetic results also verified the significantly increased blood PTX concentration and prolonged circulation of G-PTX compared with L-PTX and Abr (Abraxane) at the same dose (Supplementary Fig. S5c). Enhanced accumulation of G-PTX at the HGC-27 tumor sites (both subcutaneous xenograft and orthotopic tumors) was also imaged compared to L-PTX (Supplementary Fig. S5d, e). Similar trends were observed in three other subcutaneous tumor models (A549, MCF-7, and HGC-27/T), demonstrating a broad-spectrum tumor-targeting ability of G-PTX (Supplementary Fig. S5f–h). The tissue distribution experiment revealed significantly elevated drug content in HGC-27 subcutaneous tumor in G-PTX group compared with L-PTX, Abr and Taxol (Fig. 1i). On the other hand, the drug content in the liver, spleen and muscle tissues of the G-PTX was significantly lower than L-PTX, which demonstrated its high efficacy and low toxicity (Fig. 1i and Supplementary Fig. S6a). Meanwhile, the active tumor targeting of G-PTX was mainly achieved through GLUT1-mediated mechanism (verified through competition assay of GLUT1 specific inhibitor STF-31, and siRNA silencing), and G-PTX could be further endocytosed via both clathrin- and caveolae-dependent pathways (Fig. 1j and Supplementary Fig. S6b–f).

In vitro anti-tumor experiments showed that the IC50 values of G-PTX were more than one order magnitude higher than those of L-PTX and Abr in various tumor cell lines, especially in drug-resistant (PTX) cell lines (Supplementary Fig. S7a). As for in vivo anti-tumor assay with the initial average tumor volume of 200 mm^3^, G-PTX (30 mg PTX/kg) showed complete suppression of tumor growth on HGC-27/T (drug-resistant) tumor-bearing mice model, reducing the tumor volume to 193 mm^3^, displaying sharp contrast with Abr group (893 mm^3^) at the same dose (Fig. 1k). In HGC-27 tumor-bearing mice, two-thirds of the tumors in the G-PTX group completely disappeared at the end of experiment, while the average tumor volume in the Abr group remained 365 mm^3^, at the same dose (30 mg PTX/kg) (Fig. 1k). On several other models, G-PTX also performed significantly better than Abr (Supplementary Fig. S7b). Patient-derived xenograft (PDX) tumor models with intrinsic drug resistance were further constructed, on which G-PTX maintained its long-term suppression of tumor growth (Fig. 1l and Supplementary Fig. S7c). Besides, G-PTX proved to impede the generation of drug resistance, as the HGC-27 subcutaneous tumor remained its sensitivity to PTX after continuous low-dose stimulation by G-PTX, while significant resistance was generated after Abr treatment (Supplementary Fig. S7d). Meanwhile, further studies confirmed that G-PTX reversed the pre-existing drug resistance and prevented the development of acquired resistance by inhibiting P-glycoprotein efflux function and regulating the autophagy in tumor cells (Fig. 1m and Supplementary Fig. S8a–e).

The pre-clinical safety of G-PTX and the blank ginposome (G-Blank) were further verified in several aspects: (1) high maximum tolerable dose (MTD): the MTD of G-Blank was >400 mg/kg (ginsenoside content) and the MTD of G-PTX was >90 mg/kg (PTX content), the latter was equivalent to the MTD of Abr (Supplementary Fig. S9a). (2) low hemolysis: no evident hemolysis was observed at 1 mg/mL ginsenoside concentration (Supplementary Fig. S9b). Also, the body weight, haemocytes counts and blood biochemical indicators monitored in the in vivo efficacy experiments further confirmed the safety of G-PTX (Supplementary Fig. S10a–c).

In conclusion, based on the structural similarity between ginsenosides and cholesterol, we have innovatively constructed a novel glycosylated biomimetic L-PTX with simple formulation, high drug-loading capabilities, desirable efficacy, convenient preparation at low costs, and promising pre-clinical safety. This novel liposomal delivery technology could potentially serve as a versatile platform for various anticancer drugs, and the clinical translation of G-PTX is currently underway.

## Supplementary information

Supplemental Material
