# Supplementary material for: Identification and construction of a novel biomimetic delivery system of paclitaxel and its targeting therapy for cancer
Source: Signal Transduct Target Ther. 2021 Jan 27;6:33. doi: 10.1038/s41392-020-00390-6 (PMC7840929; doi:10.1038/s41392-020-00390-6)
Supplement: Supplementary file 1 — Supplemental Material [file 41392_2020_390_MOESM1_ESM.docx]

**Supplemental Material for**

**I****dentification and construction of a novel biomimetic delivery system of paclitaxel and its targeting therapy for cancer**

Xue Wang^1,§^, Qing Shen^4,§^, Wanwei Zheng^2,§^, Yahua Wang^1^, Yujen Tseng^2^, Zhongguang Luo^2^, Xiaoyou Wang^1^, Lei Shi^4^, Chong Li^1,3,5,*^, and Jie Liu^2,*^

^1^Medical Research Institute, College of Pharmaceutical Sciences, Southwest University, Chongqing 400715, China; ^2^Institute of Digestive Diseases, Huashan Hospital, Fudan University, Shanghai 200041, China; ^3^State Key Laboratory of Esophageal Cancer Prevention & Treatment, Zhengzhou University, Zhengzhou 450052, China; ^4^Hangzhou YITU Healthcare Technology Co., Ltd, Hangzhou 310012, China; ^5^Xiamen Ginposome Pharmaceutical Co., Ltd, Xiamen 361026, China

^*^Corresponding authors: Chong Li ([chongli@swu.edu.cn](mailto:chongli@swu.edu.cn)); Jie Liu ([jieliu@fudan.edu.cn](mailto:jieliu@fudan.edu.cn))

^§^These authors contributed equally: Xue Wang, Qing Shen, Wanwei Zheng

**This file includes:**

Materials and Methods

Figures. S1 to S10

References

# Materials and Methods

Reagents and antibodies

Lecithin and cholesterol were purchased from A.V.T. Pharmaceutical Co., Ltd. (Shanghai, China). Paclitaxel and ginsenosides Rg5, Rh3, Rb1, Rh4, F1, F2, Rf and Rg1 were provided by Xiamen Ginposome Pharmaceutical Co., Ltd (purity > 98%). 1,6-Diphenyl-1,3,5-hexatriene (DPH) was purchased from J&K Scientific Co., Ltd. NBD-PE was purchased from Shanghai Aladdin industrial Co., Ltd. GLUT1 recombinant protein was purchased from OriGene Biotechnology Co., Ltd. STF-31 was purchased from Shanghai Bide Pharmatech Co., Ltd. siRNAs were purchased from Shanghai Sangon Biotechnology Co., Ltd. The following primary antibodies were used for immunoblot and immunofluorescence with indicated dilution: rabbit anti-P62 (Abcam, ab207305, lot GR243504-9, 1:1000), rabbit anti-Beclin1 (Abcam, ab207612, lot GR310545-14, 1:2000) , rabbit anti-LC3B (Abcam, ab192890, lot GR3213821-1, 1:2000 for immunoblot, 1 µg/mL for immunofluorescence), rabbit anti-APG5L/ATG5 (Abcam, ab108327, lot GR 312199-4, 1:1000), rabbit anti-LAMP2 (Abcam, ab199946, lot GR273474-12, 1:2000; Bioss, bs2379R, lot AG09041752, 1:100 for immunofluorescence), rabbit anti-P Glycoprotein (Abcam, ab170904, lot GR217576-16, 1:1000). Beta-Actin antibody ((Protein Tech, 20536-1-AP, lot 00054123, 1:2000), mouse anti-GLUT1 (Santa Cruz, sc-377228, lot C2618, 1:100 for immunoblot, 1:50 for immunofluorescence), rabbit anti-GLUT1 (Abcam, ab115730, lot GR106925-46, 1:100000 for immunoblot, 1:100 for immunofluorescence), rabbit anti-GLUT2 (Abcam, ab192599, lot GR220364-9, 1:10000 for immunoblot, 1:100 for immunofluorescence) mouse anti-GLUT3 (Santa Cruz, sc-74497, lot K2117, 1:100 for immunoblot, 1:50 for immunofluorescence) and mouse anti-GLUT4 ( Santa Cruz, sc-53566, lot D0218, 1:200 for immunoblot, 1:50 for immunofluorescence).

Animals

All animal studies, unless otherwise stated, were performed at Southwest University with approval by

the experimental animal ethics committee of College of Pharmaceutical Sciences，Southwest University. 6- to 8-week-old Balb/c nude mice were housed in a pathogen-free facility and used to establish xenograft models. Healthy rabbits (2.0-3.0 kg) were used to carry out hemolysis assay. Sprague-Dawley rats (200-220 g) were used in the pharmacokinetic evaluation of G-PTX.

Cell lines

The human gastric cancer cell line, HGC-27, was purchased from Procell Life Science & Technology and cultured in RPMI 1640 medium. MCF-7 cells and A549 cells (Procell Life Science & Technology) were cultured in MEM medium containing bovine insulin and Ham's F-12K medium, respectively. HGC-27/PTX cell line was established by culturing HGC-27 cells with paclitaxel in a dose-escalation manner^1^ until reaching a concentration of 200 ng/mL. HGC-27/T and A549/T cells (Procell Life Science & Technology) were cultured in RPMI 1640 medium with 200 ng/mL paclitaxel and Ham's F-12K medium with 500 ng/mL paclitaxel, respectively. All the above cells were cultured in complete medium containing fetal bovine serum (FBS). HGC-27, MCF-7, A549 cell lines were authenticated using Short Tandem Repeat (STR) analysis. 3T3-L1 cells, purchased from KeyGen Biotech, were cultured in DMEM medium with 10% calf serum. RAW 264.7 cells, obtained from the Cell Bank at the Chinese Academy of Sciences, were cultured in DMEM medium with 10% FBS.

Preparation and characterization of ginposomes and control liposomes

The formulations were prepared via thin film hydration. Compositions of the prepared formulations were as follows: (1) G-PTX (paclitaxel-loaded ginposome), lecithin/Rg5/PTX (mass ratio, 10:4:1); (2) G-Blank (blank ginposome without paclitaxel), lecithin/Rg5 (mass ratio, 10:4); (3) L-PTX (paclitaxel-loaded liposome), lecithin/cholesterol/PTX (mass ratio, 10:2:1). All the components of ginposomes could be dissolved in anhydrous alcohol and were dried by vacuum rotary evaporation to remove the alcohol. After a thin film layer was formed, it was hydrated with ultrapure water and then sonicated and membrane-extruded (pore size = 100 nm) to form ginposomes with controlled particle size. The mean diameter and zeta potential of the formulations were measured with a DLS analyzer (Malvern ZetasizerNano ZS90). The morphology of ginposome was examined with a transmission electron microscope (FEI Tecnai G20, USA). As control liposomes, L-PTX were prepared using a similar method as described above, except that the organic solvent was trichloromethane: ethanol (1:1, v/v).

To determine the encapsulation efficiency (EE) of paclitaxel, as previously described,^2^ the ginposomes were centrifuged at 11,000 *g* for 10 min at 4 °C to separate any free PTX. After centrifugation, the obtained ginposome was dissolved with methanol, and the amount of loaded PTX was determined by HPLC (Shimadzu, Kyoto, Japan) using a mixture of methanol, water, and acetonitrile at a ratio of 23:41:36 (v/v) as the mobile phase, and the detection wavelength was 227 nm. The encapsulation efficiency was calculated from the content ratio between the encapsulated PTX and its total input amount in the fabrication.

Molecular dynamics simulation

The 3D coordinate of PTX was downloaded from [www.pdb.org](http://www.pdb.org) (PDB ID:TA1). The 2D structures of Rg5, Rh3 and Rh4 were downloaded from Pubchem database, and were transformed to 3D structure using small molecule module in Schrodinger2015. The 3D structures of these small molecules were optimized using Gaussian09 with ab initio Hartree Fock calculations at the 6-311G* level. The Quantum Mechanical Calculation generated the RESP (restrained electrostatic potential) charge of atoms of these small molecules. With the RESP charge, the force field parameters of these small molecules for molecular dynamic simulation were generated using antechamber package in AmberTools^3,4^ and ACPYPE (AnteChamber PYthon Parser interfacE) script.

The CHL-POPC bilayer membrane systems consisted of 320 POPC and 128 cholesterols, and were built with membrane builder in CHARMM-GUI web-server.[^5-7^](#_ENREF_1) The coordinates of CHL were replaced with Rg5, Rh3 or Rh4 to build the structural model of Rg5-POPC, Rh3-POPC or Rh4-POPC bilayer systems. Another CHL-POPC bilayer membrane system was generated with membrane builder in CHARMM-GUI web-server for the preparation of PTX-CHL-POPC bilayer membrane system. 32 PTX molecules were inserted at the lattice point of the liposome membrane system. The POPC and CHL molecules which had a distance of < 1.8 Å with PTX were deleted. The final model of the liposome membrane system consisted of 320 POPC, 128 CHL and 32 PTX. The ratio of PTX: CHL: POPC was 1:4:10. Water molecules and NaCl at physiological concentration (125 mM) was also added to the membrane systems. For the preparation of PTX-Rg5-POPC system, the structure model of Rg5 was superimposed to the skeleton of CHL, then the CHL molecules in PTX-CHL-POPC membrane model were replaced by Rg5. The PTX-Rh3-POPC and PTX-Rh4-POPC bilayer systems were built through the same process.

The CHARMM36 force field was used for POPC, and the CHARMM TIP3P model was used for water. The CHARMM36 force field parameters of paclitaxel and Rg5 were prepared. The MD simulations were carried out using GROMACS2018.^8,9^ NPT ensemble was applied for the simulation with the Nose-Hoover thermostat to keep the temperature at 310 K and the Parrinello-Rhaman method to maintain a constant pressure of 1 bar. All bonds were constrained by the LINCS algorithm. Electronic interactions were calculated using the Partical-Mesh Ewald (PME) algorithm. The cutoff for van der Waals interactions was 12 Å with a smooth switching at 10 Å used to truncate the van der Waals potential energy at the cutoff distance. 200-ns dynamic simulations were carried out for all the bilayer systems with coordinates saved at every 2 ps for later analysis.

Molecular docking

The GLUT1 structure mouse models were built by SWISS-MODEL server using homo GLUT3 in an outward-open state (4wcz) as template.^10^ Models were built based on the target-template alignment using ProMod3 in SWISS-MODEL. The sequence identity between the template homo GLUT3 sequence and mouse GLUT1 sequences was 67.03%. Coordinates which were conserved between the target and the template were copied from the template to the model. Insertions and deletions were remodeled using a fragment library. Side chains were then rebuilt. Finally, the geometry of the resulting model was regularized by using a force field. If loop modeling with ProMod3 fails, an alternative model was built with PROMOD-II.^11^ The global and per-residue model quality was assessed using the QMEAN scoring function.^12^ For improved performance, weights of the individual QMEAN terms had been trained specifically for SWISS-MODEL.

We prepared ginsenoside Rg5 structure with known structures presented in the PubChem. The structure was generated by the two-dimensional Sketcher in Maestro, and was converted to three-dimensional structure. The structure model of disaccharide fragment was derived from three-dimensional structure of ginsenoside Rg5 by cutting the atoms in Rg5 which did not belong to the disaccharide fragment. The glucose structure was downloaded from pubchem database. The ligand conformation of disaccharide and glucose for docking were prepared with the LigPrep module, and the pKa of the ligands was calculated using the Epik module. The structure models of mouse GLUT1 were preprocessed with the Protein Preparation Wizard, bond orders were assigned, hydrogens were added, and the H-bond network was optimized. The structure model of mouse GLUT1 was energy-minimized using the OPLS 2005 force field. The Molecular Docking was performed using Glide in the Schrodinger software suite.^13-15^ No restraints were used during docking. Computational docking was performed by the Glide module in extra-precision mode and default values for grid generation.

Fluidity measurements of the vesicle membrane

The membrane fluidity of blank vesicles containing different ginsenosides was examined as previously described.^16^ Ginsenoside-anchored vesicles and control liposomes were labeled with DPH at a molar ratio of DPH: lecithin (1:200). Fluorescence anisotropy of DPH measurements was performed in a microplate reader (Tecan, Switzerland). Excitation and emission wavelengths of DPH were set at 360 nm and 430 nm, respectively. The changes of DPH anisotropy values of the vesicles were examined under a temperature gradient from 20-50 ℃.

The membrane fluidity of PTX-loaded formulations containing different molar ratios of Rg5 to PTX was examined. The PTX-loaded formulations containing a series of molar ratios of Rg5 to PTX was prepared first, and their DPH fluorescence anisotropy values were measured at a fixed temperature of 37 ℃. The fluorescence anisotropy (*r*) was determined according to the following equation: *r* = (*I*_‖_ – *I*_⊥_)/( *I*_‖_ + 2*I*_⊥_), where the *I*_‖_ and *I*_⊥_ are the fluorescence intensities of the emitted light polarized parallel and perpendicular to the polarized light of excitation.

Fluorescence recovery after photobleaching (FRAP) assay of the vesicle membrane

FRAP assay was performed as previously described.^17^ NBD-PE was labeled on the formulations at a molar ratio of NBD-PE: lecithin (1:100). The obtained liposomal suspension was sized by sonication. Unincorporated NBD-PE was removed by gel filtration (Sephadex G-25). Excitation and emission wavelengths of NBD-PE were 463 nm and 534 nm, respectively. FRAP measurements were carried out with a Nikon A1 confocal microscope (Nikon, Japan). The region of interest in liposome was photobleached using the argon ion laser at 488 nm and 100% intensity for a period of 400 ms. Immediately after bleaching, fluorescence intensities of the bleached spots were monitored for 280 s with 10-s intervals at low excitation energy, and the recovery curves of the fluorescence were obtained. For normalization of FRAP data, the fluorescence signal in a region of interest was measured according to the following equation: *R* = (*I*_t_ – *I*_0_)/(*I*_pre_ – *I*_0_), where *I*_t_ is the average intensity in the bleached region at any time-point after bleaching, *I*_pre_ is the average intensity in the region of interest before bleaching, *I*_0_ is the average intensity in the region of interest immediately after photobleaching.

Freeze-drying of G-PTX

The formulation containing a small amount of lyoprotectant (trehalose) was quickly frozen to -50 ℃ and kept for 4 h, then the temperature was elevated to -30 ℃ in 2 h and kept for 50 h. Afterwards, the temperature was elevated to -20 ℃ in 1 h, then kept for 5 h. The temperature was elevated to 20 ℃ in 3 h and then kept for 6 h. The whole process was carried out under a vacuumed pressure of 10 pa. The lyophilized samples were re-suspended with ultrapure water to the initial lipid concentrations at room temperature. Then, the mean diameter and drug encapsulation efficiency of the formulations were measured. To evaluate the drug retention during lyophilization processes, G-PTX was treated by centrifugation at 11,000 *g* for 10 min to separate the free PTX. The content of the drug in the supernatant was measured with HPLC. To evaluate the stability of the lyophilized liposome during storage, the mean diameter of lyophilized liposome was measured once a week for 5 successive weeks.

*In vitro* drug release assay

The *in vitro* release pattern of PTX from G-PTX was studied using the dialysis bag method.^18^ G-PTX formulations containing 1 mg PTX were put into a dialysis bag (MWCO 8,000 -14,000 Da), which was immersed in 20 mL of sodium salicylate solution (1.0 M), and the samples were kept under continuous shaking at 37 ℃. At specified time points, the whole volume of sample medium was collected and replaced with an equal volume of fresh medium. The amount of PTX was measured using HPLC. All the operations met the sink conditions and were carried out in triplicate. To investigate the release mechanism of PTX from G-PTX, the results of *in vitro* release study were examined in accordance to zero-order kinetics, first order kinetics, Higuchi model, and Korsmeyer–Peppas model.

Surface plasma resonance (SPR) analysis

GLUT1 proteoliposomes were generated as reported previously.^19,20^ Conventional liposomes were destabilized by 1 mM Triton X-100 and mixed with GLUT1 recombinant protein (100:1, w/w) in 100 mM KH_2_PO_4_ buffer (pH 7.5), 200 mM glucose, 20% (v/v) glycerol. Then, SM2 BioBeads were added to remove the detergents. The proteoliposomes were collected by ultracentrifugation at 200,000 *g* for 1 h. The sensor chip LIP-1 (Nicoya life science) was immersed in HBS buffer and briefly cleaned before assembly. The sensor chip was installed in the flow cell and activated with 20 mM CHAPS. Proteoliposomes were loaded onto the SPR sensor surface, and different concentrations of ginsenoside samples were injected and tested.

Preparation and analysis of protein corona complexes

Preparation of protein corona complexes was carried out as previously described.^21^ G-PTX and L-PTX were incubated with mouse serum (volume ratio, 1:1) at 37 ℃ for 1 h. After incubation, formulations with proteins were centrifuged at 16,000 *g* for 20 min. The acquired formulation-protein corona complexes were washed with PBS for 3 times to remove the unbound proteins. Proteins adsorbed on the formulations were analyzed by SDS-PAGE. After staining with Coomassie Brilliant Blue, the gels were rinsed by pure water and captured by Amersham imager 600 (GE).

Cellular uptake

Tumor cells were seeded onto 24-well plates. Following overnight attachment, cells were cultured in fresh glucose-free medium with or without 25 mM glucose (DMEM medium) or 25 mM sophorose and incubated 24 h. Then the cells were treated with G-PTX (molar ratio, 4:1 or 3:1) for 2 h at 37 ℃. After being rinsed 3 times with PBS, cell samples were trypsinized and centrifuged, then resuspended in PBS and analyzed by flow cytometry.

RAW [264.7](https://wenku.baidu.com/view/17abbdda4028915f804dc2af.html) macrophage cells were cultured in 24-well plates and treated with Nile red-labeled formulations for 2 h at 37 ℃. The uptake of various paclitaxel formulations by RAW [264.7](https://wenku.baidu.com/view/17abbdda4028915f804dc2af.html) cells was analyzed quantitatively by flow cytometry.

*In vitro* targeting mechanism study of ginposome

Firstly, the GLUTs 1-4 expression of HGC-27 cells was examined by immunofluorescence. Cells were grown in 24-well plates, fixed with 4% phosphate-buffered paraformaldehyde, washed with PBS, and blocked in PBS with 5% bovine serum albumin (BSA). GLUTs 1-4 antibodies were then added and the cells were further incubated at 4 ℃ overnight. After washing with PBS for three times, the cells were incubated with secondary antibodies at room temperature for 1 h, and then analyzed by fluorescence microscope.

HGC-27 cells were treated with STF-31 at a final concentration of 5 μM at 37 ℃. Subsequently, Nile red-labeled G-PTX and L-PTX were added to the cells and further incubated for 2 h. At the end of the incubation, cells were washed with ice-cold PBS thrice and fixed with 4% paraformaldehyde for 15 min. After being stained with DAPI for 5 min, cells were observed with fluorescence microscope.

The siRNAs used were as follows: GLUT1-targeting siRNA (sense sequence: 5’-GGA AUUCAAUGCUGAUGAUTT-3’); GLUT2-targeting siRNA (sense sequence: 5’-ACCAAUUCCA GCUACCGACTT-3’); GLUT3-targeting siRNA (sense sequence: 5’-GCUCUUUCCAAUUUGGC UATT-3’); GLUT4-targeting siRNA (sense sequence: 5’-AAAUUGUAGCUCUGUUCAAUC-3’). siRNAs were transfected into HGC-27 and MCF-7 cells. When the density of the cells reached 70 %, each well was added with medium without antibiotics and serum. A mixture of siRNA and transfection reagent were incubated with the cells for 6 h. Then the medium was replaced with fresh medium containing 10% serum for 16 h of incubation. After the gene expression was suppressed by siRNA, the protein content of GLUT1, GLUT2, GLUT3, and GLUT4 were quantified by western blot for verification. Then the uptake of G-PTX was tested on the transfected cells. The transfected HGC-27 and MCF-7 cells were incubated with Nile red-labeled G-PTX for 2 h at 37 ℃. The amount of fluorescein ingested by the cells was imaged with fluorescence microscope or quantified by flow cytometry.

Endocytic pathways

The cells were treated with endocytic pathway inhibitors including chlorpromazine (CPZ), filipin, amiloride and M-β-CD. After being saturated with the inhibitors for 30 min at 37 ℃, the cells were incubated with Nile red-labeled G-PTX or L-PTX for 2 h. The fluorescence intensity of the cells was identified with flow cytometry.

*In vitro* cytotoxicity

The *in vitro* cytotoxicity of G-PTX was determined by MTT assay.^22^ HGC-27, HGC-27/T, A549, A549/T and MCF-7 cells were seeded in 96-well plates (1×10^4^ cells/well). Following overnight attachment, the cells were treated with free PTX, free Rg5, free Rg5 + free PTX, Abr (Abraxane), L-PTX, G-PTX or G-Blank for 48 h. The PTX concentrations ranged from 0 to 100 μg/mL, and at every concentration, the ratio of Rg5/PTX consistently maintained at 4:1 (mass ratio). The culture media was then removed and replaced with fresh complete medium containing MTT solution (final concentration was 0.5 mg/mL). Following incubation for 4 h, the medium was removed, and the purple formazan crystals were dissolved in 150 μL of DMSO. The absorbance of each well was measured at 490 nm using a microplate reader (Bio-Rad, USA). IC50 values were calculated by the GraphPad Prism 6 software.

Cellular uptake of Rh123

To assess the effect of G-PTX on the functional activity of P-gp, the accumulation of Rh123 in HGC-27 or HGC-27/T cells were detected by flow cytometry. Cells were treated with G-PTX with 40 μg/mL final concentration of Rg5 or 10 μg/mL verapamil for 1 h at 37 ℃. Subsequently, Rh123 at 5 μg/mL was added to the cells and further incubated for 1 h. At the end of the incubation, cells were washed with PBS for three times and analyzed using flow cytometry.

Cellular accumulation of paclitaxel

HGC-27 and HGC-27/T cells were seeded in 24-well plates at a density of 2×10^5^ cells/well and incubated overnight. HGC-27 cells were treated with G-PTX and L-PTX in culture medium with equivalent PTX concentration of 0.5 μg/mL, and incubated at 37 ℃ for 2 h. HGC-27/T cells were treated with formulations equivalent to PTX concentration of 10 μg/mL. Verapamil was used as a positive control. After treatment, cells were lysed in RIPA buffer containing protease inhibitors. The total cellular protein content of the cells was quantified by BCA protein assay kits. The concentration of PTX was measured by HPLC. All experiments were conducted in triplicate.

Orthotopic tumor implantations

Orthotopic tumor transplantation was performed as previously reported.^23^ Nude mice bearing s.c. HGC-27 tumors were resected aseptically to acquire tumor pieces. An incision was made on the stomach wall of the healthy nude mouse, and then a tumor piece of 150 mg was fixed in the pocket of the serosal surface which was closed with absorbable sutures. The abdominal wall was also closed with absorbable sutures. All procedures were performed in a sterile environment. Furthermore, 21 days after orthotopic tumor was transplanted，the stomach tissues were processed for histological examinations.

*In vivo* imaging

Four types of subcutaneous tumor models (HGC-27, HGC-27/T, A549, and MCF-7 cells) and the HGC-27 orthotopic xenograft models were used in this assay. For s.c. models, the tumor had grown to ~150 mm^3^ for the experiment; for orthotopic models, the tumor was transplanted 21 days before the experiment. A proper amount of IR-783 labeled liposomes were intravenously administrated. The mice were imaged through intravital imaging (FX-Pro, Carestream) at 1, 2, 4, 6, 8 and 12 h post-injection, using the excitation wavelength of 720 nm and the emission wavelength of 790 nm. After 12 h, the mice were sacrificed and the major organs (brains, hearts, lungs, livers, spleens, and kidneys) and tumors were collected for *ex vivo* imaging.

Immunofluorescent staining of tumor sections

Nile red-labeled G-PTX or L-PTX was intravenously injected into the HGC-27 tumor-bearing mouse models. After 4 hours of administration, the tumor was removed and embedded in OCT (Tissue Tek). Sections with a thickness of 5 μm were obtained with a Microtome-Cryostat (Leica, Germany), collected on glass slides and fixed in 4% phosphate-buffered paraformaldehyde. The slides were blocked in PBS with 5% BSA at room temperature for 1 h, then incubated overnight at 4 ℃ with anti-GLUT1 primary antibody. After washing with PBS three times, the slides were incubated with appropriate secondary antibodies conjugated to Alexa fluor 488 at room temperature for 1 h, and then stained with DAPI for 5 min. The slides were imaged with fluorescence microscope.

Pharmacokinetics and tissue distribution study

To investigate the pharmacokinetics of PTX, formulations containing PTX were intravenously injected to rats at an equivalent dose of 15 mg/kg PTX. At time points of 5, 15, 30, 45 min, 1, 2, 4, 6, 8, 12 and 24 h post-injection, blood samples (approximately 0.2 mL) were collected in heparinized centrifuge tubes. Then the blood was immediately centrifuged at 1500 *g* for 10 min. The plasma was obtained and stored at -80 ℃ before HPLC analysis. To examine the biodistribution, mice bearing HGC-27 tumors were administrated with PTX formulations at a dose of 15 mg/kg PTX via tail vein injection. The mice were sacrificed at predefined time periods (2, 4, 8, and 12 h). The major organs, muscle and tumor were excised and thoroughly rinsed with ice-cold saline, dried and weighted.
 The tissues collected were homogenized in saline. Aliquots of 50 μL of plasma or 200 μL of tissue homogenate were mixed with 4 mL tert-butylmethylether containing 20 μL of norethindrone (internal standard). The samples were extracted on vortex-mixer for 2 min and then centrifuged (6000 *g*, 10 min). The supernatant (tert-butylmethylether) was transferred to a clean tube and dried under a gentle stream of nitrogen. The acquired plasma and tissue residue were re-dissolved in acetonitrile, then centrifuged at 11,000 *g* for 10 min. The obtained supernatant was injected into HPLC for analysis.

*In vivo* tumor regression experiments and safety evaluation

Balb/c nude mice were inoculated subcutaneously with a sufficient amount (5×10^6^) of cells (HGC-27, HGC-27/T, A549, A549/T or MCF-7 cells). When the tumor volume reached ~200 mm^3^, mice were randomly divided into 8 groups and treated weekly with saline, G-Blank, 15 mg/kg Taxol, 15 mg/kg L-PTX, 30 mg/kg G-PTX, 15 mg/kg G-PTX, 30 mg/kg Abraxane or 15 mg/kg Abraxane for 4 weeks. The tumor dimensions and body weight were measured twice a week, and the tumor volume was calculated by the following formula: volume (mm^3^) = length × (width)^2^ × 1/2. Tumors were measured for 28 days, then the mice were sacrificed. The blood of the mice after treatment was collected. Mice with tumors exceeding 1800 mm^3^ were euthanized in this work. The quantities of alkaline phosphatase, aspartate transaminase, and bilirubin of different mice were determined using the corresponding assay kits. The amount of platelet and neutrophil of whole blood in different mice were detected by whole blood analyzer.

PTX sensitivity assay of the tumor after treated by paclitaxel formulations

HGC-27 human gastric cancer xenograft models were established and treated as previously described with minor modifications.^24^ Briefly, HGC-27 tumor bearing-mice were treated twice a week with saline, G-Blank at a dose of 40 mg/kg Rg5, G-PTX and Abraxane at a dose of 10 mg/kg PTX for 4 weeks. Tumor tissues were then collected and cut into fragments. The fragments were digested with collagenase at 37 ℃ for 1 h in incubator shaker. The sample was filtrated and washed twice with PBS to form cell suspensions, and then cultured in complete medium. The sensitivity of the cells to paclitaxel was determined by MTT assay.

Establishment of human gastric patient-derived xenograft (PDX) tumor model

Nude mice were used to generate subcutaneous PDX tumor models,^25,26^ human gastric cancer tissues were obtained from two gastric cancer patients at Fudan University of Huashan Hospital in accordance with a protocol approved by its institutional review board. Briefly, nude mice were implanted subcutaneously with fresh tumor tissue fragments for the first murine generation. When the tumor volume reached about 1000-1500 mm^3^, the mice were sacrificed, and the tumor fragments were then harvested and re-transplanted to nude mice for subsequent generations. For *in vivo* tumor regression experiments, the tumor specimens were inoculated into the right flank of each mouse. When tumor volumes reached ~200 mm^3^, nude mice were divided into six groups randomly, and received different treatments including: (1) saline, (2) G-Blank, (3) 30 mg/kg Abraxane, (4) 15 mg/kg Abraxane, (5) 30 mg/kg G-PTX and (6) 15 mg/kg G-PTX. Tumor-bearing mice were administered once a week and the tumor dimensions and body weight were measured twice a week. At the end of the experiment, mice were killed and tumors were excised and photographed.

Western blot assay

The cell suspensions were lysed in RIPA buffer containing 1 mM PMSF. The protein concentration was quantified by BCA protein assay kits. The samples were resolved on SDS-PAGE gels and transferred onto PVDF membranes. The membranes were blocked in Tris-buffered saline with 0.1% Tween-20 supplemented with 5% non-fat milk at room temperature for 1 h, and incubated with primary antibodies. Bound proteins were detected with HRP-conjugated secondary antibodies and ECL reagents.

Double-stained immunofluorescence for autophagy

HGC-27/T cells were incubated with G-PTX and L-PTX at doses of 10 μg/mL PTX or 10 μg/mL chloroquine (CQ). After treatment, cells were washed with PBS (pH 7.4) and fixed with 4% paraformaldehyde. Then the cells were permeabilized with PBS containing 1% BSA and 0.2% Triton X-100 for 10 min, and blocked in PBS with 5% BSA at room temperature for 1 h. Cells were incubated with primary antibody against LC3 overnight at 4 °C, washed with PBS and incubated with Alexa Fluor-conjugated secondary antibody for 1 h. After that, cells were incubated with another primary antibody against lysosomal-associated membrane protein-2 (LAMP-2), washed with PBS and incubated with Cy3-conjugated secondary antibody for 1 h. After washed with PBS for three times, cells were stained with the nuclear dye DAPI in PBS for 5 min. The cells were imaged with a high-content imaging device (Operetta CLS, Perkin-Elmer, USA).

Maximum tolerated doses (MTD) assay

The MTD of paclitaxel formulation was assessed using a dose-escalation method on healthy nude mice as reported previously.^27^ G-PTX or Abr was delivered by i.v. injection at doses of 30, 50, 70 and 90 mg/kg (PTX equiv.), and G-Blank was delivered by the tail vein injection at doses of 100, 200, 300 and 400 mg Rg5/kg. The survival and body weight of each mouse was observed for two weeks. The MTD was defined as no mice displayed more than 10% of body weight loss, and neither death nor abnormal behavior including the inability to eat and drink occurred within 2 weeks.

Hemolysis assay

The rabbit blood was freshly obtained and centrifuged at 800 *g* for 5 min to collect precipitated RBCs. Then, 1 mL of RBCs sample was added to 10 mL of physiological saline and purified by centrifugation at 800 *g* for 10 min. The RBCs were further washed twice with 10 times volume of physiological saline and then diluted 50 times with physiological saline. For hemolysis investigation, 2.5 mL RBCs suspension was incubated with G-PTX, L-PTX, and Abr at a final concentration of 250 μg/mL PTX, and G-Blank at a final concentration of 1 mg/mL Rg5 with a total volume of 5 mL. The negative control group and the positive control group consisted of 2.5 mL RBCs suspended in saline and ultrapure water, respectively. After incubation at 37 ℃ for 3 h, the samples were centrifuged at 800 *g* for 5 min, and then photographed. The percentage of hemolysis was calculated as follows equation：Hemolysis (%) = (*A*_sample_ – *A*_negative_)/ (*A*_positive_ – *A*_negative_) ×100, where *A*_sample_ is the absorbance of the sample, *A*_negative_ and *A*_positive_ are the absorbance of the negative control group and the positive control group, respectively.

Statistical analysis

Statistical analyses were performed with GraphPad Prism 6 software. Data were expressed as the mean ± s.d. Animals were randomized before the initiation of treatment. Data were analyzed by one-way ANOVA or two-way ANOVA for multiple-group comparison. The two-tailed unpaired Student’s *t*-test was used to evaluate the differences between the two groups. *P* values of less than 0.05 and 0.01 were regarded as significant and very significant.

# Figure S1


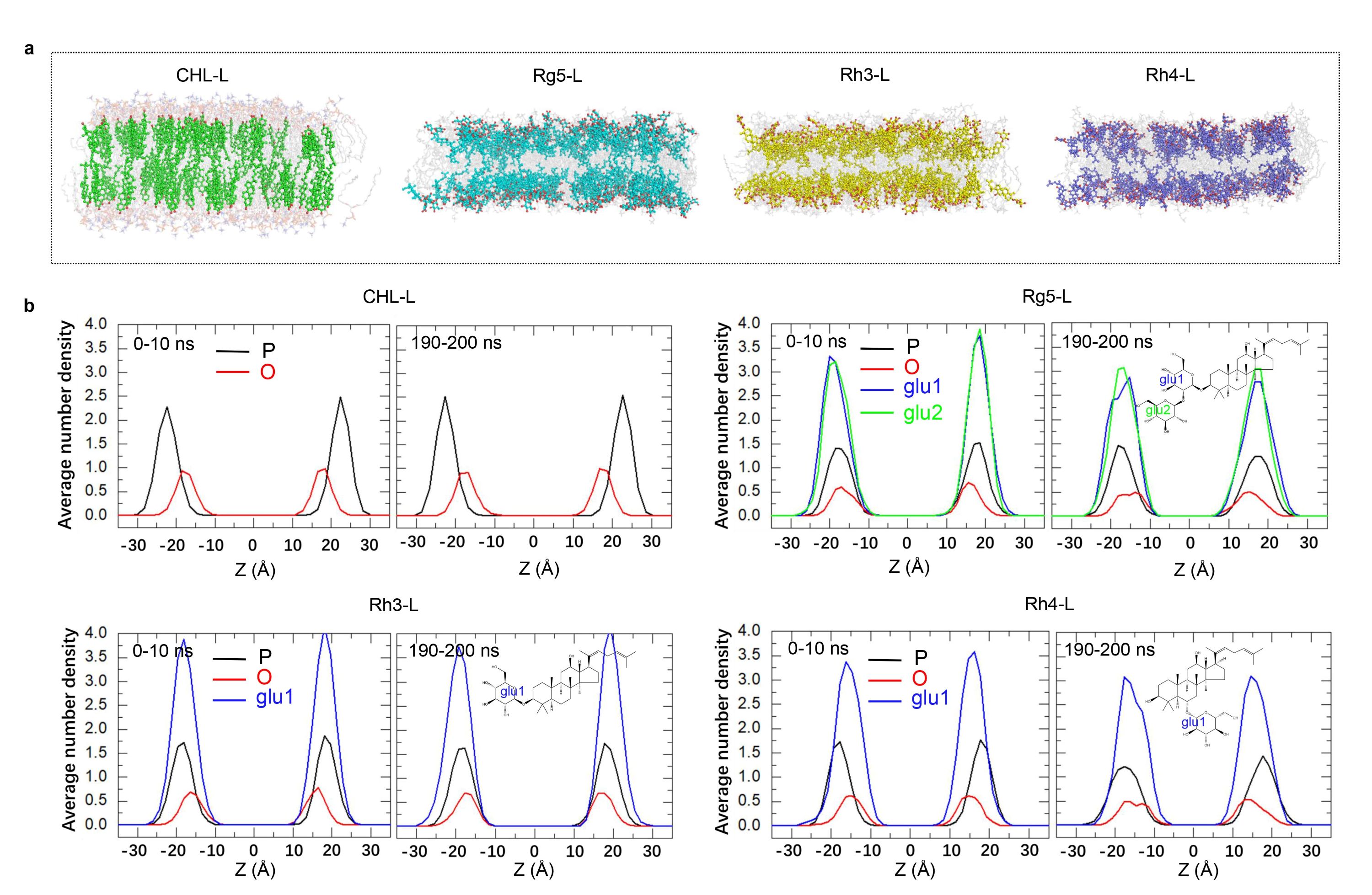


**Fig. S1 Molecular dynamic simulations of ginsenoside-anchored liposomes and control liposomes. a** The snapshots of Rg5, Rh3, Rh4 and cholesterol (CHL) anchored in the membrane bilayer of POPC liposomes (Rg5-L, Rh3-L, Rh4-L, CHL-L) at 200 ns in molecular dynamic simulation. POPC (white), CHL (green), Rg5 (blue), Rh3 (yellow) and Rh4 (purple-blue) are shown in stick. **b** Average number density of the membrane models at the first and last 10 ns of the 200 ns simulation. In Rg5-L, Rh3-L and Rh4-L, the phosphorus atom in POPC, the oxygen atom connecting the glycosyl and skeleton, and the first glucose unit conjugated to the skeleton are colored in black, red, and blue, respectively. The second glucose unit in Rg5-L is colored in green. In CHL-L, the phosphorus atom in POPC and oxygen atom in CHL are colored in black and red, respectively. In amphipathic lipid bilayers, the phosphorus atoms in the hydrophilic head of POPC represented the water-oil boundary between the hydrophobic and the aqueous regions. According to the distribution of different structures along the Z-axis depicted by their average number densities, the oxygen atoms in cholesterol distributed closer to the hydrophobic center of the POPC membrane compared with the phosphorus atoms, indicating that most oxygen atoms in cholesterol could not reach the aqueous phase. In contrast, the distribution of the glycosyls in Rg5 and Rh3 was similar to the phosphorus atoms, indicating that these glycosyls could reach the aqueous phase. Most of the glycosyls in Rh4, however, distributed closer to the hydrophobic center of the POPC membrane than the phosphorus atoms, indicated less exposure on the membrane surface. Meanwhile, this location suggested more interactions between the glycosyls in Rh4 and the POPC membrane, resulting in lowered membrane stability of Rh4-L compared with Rg5-L and Rh3-L systems.

# Figure S2


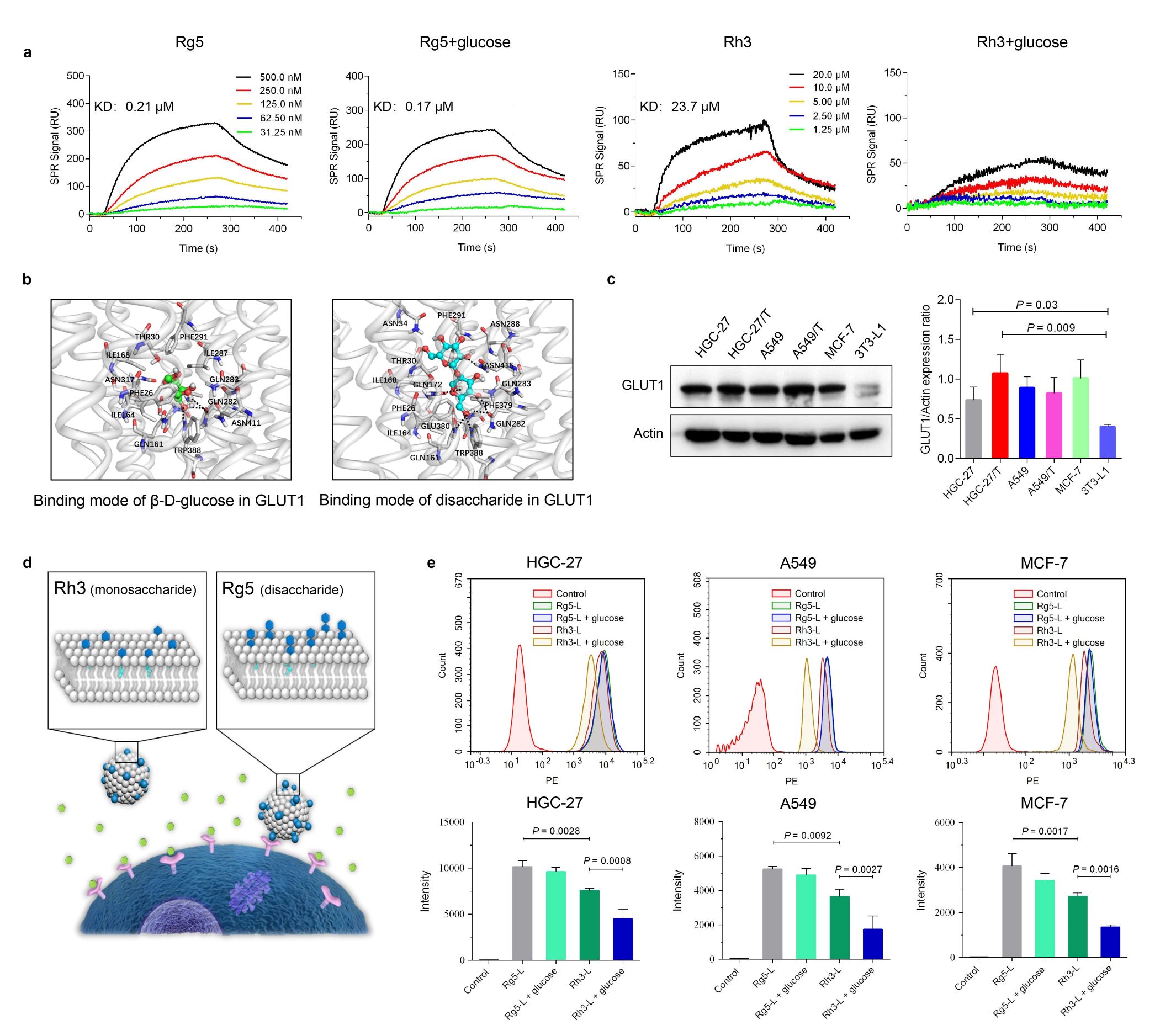


**Fig. S2 Further selection of ginsenosides by target-binding affinity and *in vitro* targeting ability: the comparison between Rg5 and Rh3. a** The binding affinity between Rg5, Rh3 and GLUT1, with or without 25 mM free glucose. Compared with Rh3 monosaccharide glycosyl, Rg5 exhibited a 110-fold-stronger affinity with GLUT1 due to its disaccharide glycosyl. Free glucose significantly reduced the affinity of Rh3 with GLUT1, yet its influence on Rg5 was negligible. **b** The binding modes of β-D-glucose derived from Rh3 (green) and the disaccharide derived from Rg5 (blue) in GLUT1 by molecule docking. The GLUT1 protein is shown in white, the residues involved in binding are represented by sticks, and the hydrogen-bond networks are denoted by black dashed lines. In the binding pocket of GLUT1, the β-D-glucose formed hydrogen bonds with GLN282, GLN283 and TRP388, while the disaccharide could form more hydrogen bonds with GLUT1, while GLN282, TRP388, GLN161, GLN172 and ASN415 were all involved in the hydrogen bond network. The calculated ΔG of β-D-glucose and disaccharide for binding with GLUT1 were -5.88 kcal/mol and -8.76 kcal/mol, indicating that the binding affinity of disaccharide was 130-fold higher than β-D-glucose, a similar trend with the result of the SPR assay. **c**, The GLUT1 protein expression in a variety of tumor cells by western blot (n = 3; two-tailed *t*-test). **d**, **e** The targeting ability of Rh3-anchored liposome was significantly interfered by free glucose at high concentration (25 mM), while Rg5-anchored liposome maintained its targeting ability, shown by schematic diagram (**d**) and quantitative results of cellular uptake by flow cytometry in three different cell lines (**e**). n = 3. *P* values were determined by one-way ANOVA. Data were expressed as mean ± s.d.

# Figure S3


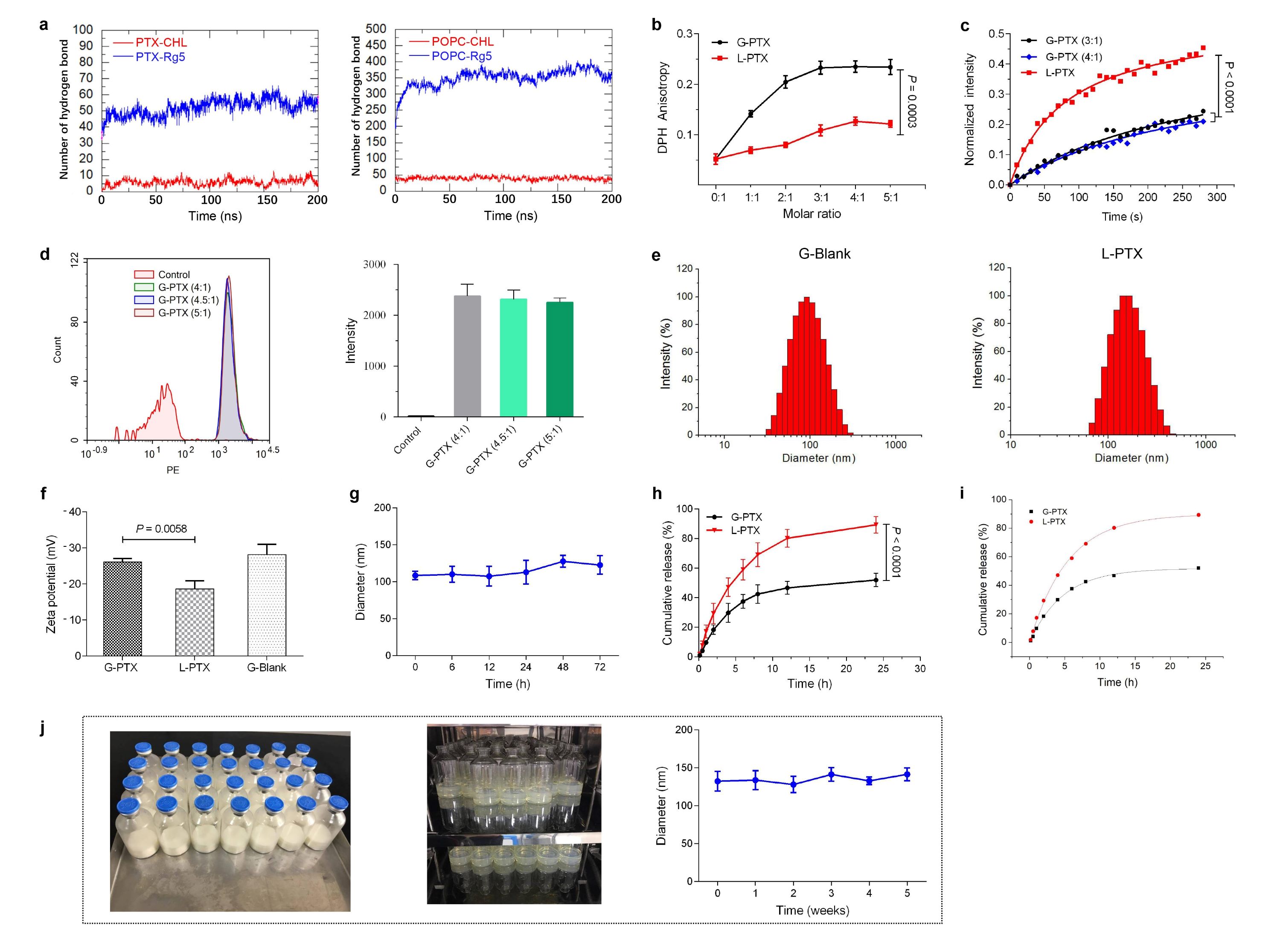


**Fig. S3 Formulation design and characterization of drug-loaded ginposomes. a** Number of hydrogen bond between different portions in G-PTX and L-PTX bilayer membrane. The number of hydrogen bond between CHL/Rg5 and PTX (left), and the number of hydrogen bonds between CHL/Rg5 and POPC (right). **b** The influence of ginsenoside Rg5 and cholesterol at different concentrations on membrane fluidity. The molar ratio of Rg5 and paclitaxel was 0:1, 1:1, 2:1, 3:1 4:1 and 5:1, respectively. The fluorescence anisotropy increased steadily from 0.052 to 0.233 when the molar ratio of Rg5 and paclitaxel was increased to 3:1, indicating a more orderly arrangement of the membrane bilayer. The anisotropy values of G-PTX with different Rg5/PTX ratios were all higher than that of L-PTX containing cholesterol at corresponding CHL/PTX molar ratios, suggesting G-PTX was more stable than L-PTX (n = 3; two-tailed *t*-test). **c** The effects of cholesterol and Rg5 on the fluidity of drug-loaded lipid bilayers measured by FRAP assay. Normalized fluorescence recovery of NBD-PE probe in G-PTX group was obviously slower than L-PTX, reflecting reduced membrane fluidity and increased stability (n = 3; two-way ANOVA). **d** The uptake of G-PTX with different molar ratios of ginsenoside and paclitaxel (4:1, 4.5:1, and 5:1) by HGC-27 cells. The further increase of Rg5 content in G-PTX showed no significant influence on its cellular uptake (n = 3; one-way ANOVA). **e** Hydrodynamic size distribution of G-Blank and L-PTX. The average particle sizes of G-Blank and L-PTX were 90 nm and 147.3 nm, respectively. **f** Zeta potential of G-Blank, G-PTX, and L-PTX (n = 3; two-tailed *t*-test). **g** Stability test of G-PTX. The mean particle diameter of G-PTX showed no significant change for 72 h at 4 ℃ (n = 3). **h** *In vitro* release profiles of PTX from G-PTX and L-PTX in sodium salicylate solution (1.0 M). G-PTX showed an obvious trend of sustained drug release compared to that of conventional liposomes (n = 3; two-way ANOVA). **i** Release curves of G-PTX and L-PTX after fitting first order kinetic model. The maximum R^2^ close to 1 indicated first order kinetic model as the best fit model for G-PTX and L-PTX. In addition, Korsmeyer–Peppas model was also used to detect the release mechanism of G-PTX. The exponent value (n) of G-PTX was lower than 0.45 (0.44), while that of L-PTX was higher than 0.45 (0.46), indicating that drug release from G-PTX and L-PTX was by quasi-Fickian diffusion mechanism and non-Fickian diffusion mechanism, respectively. **j** Photograph of G-PTX after lyophilization and hydrodynamic size of redissolved G-PTX was measured. G-PTX could be freeze-dried into powder form and the particle size was minimally altered after redissolution (n = 3). All data are presented as mean ± s.d.

# Figure S4


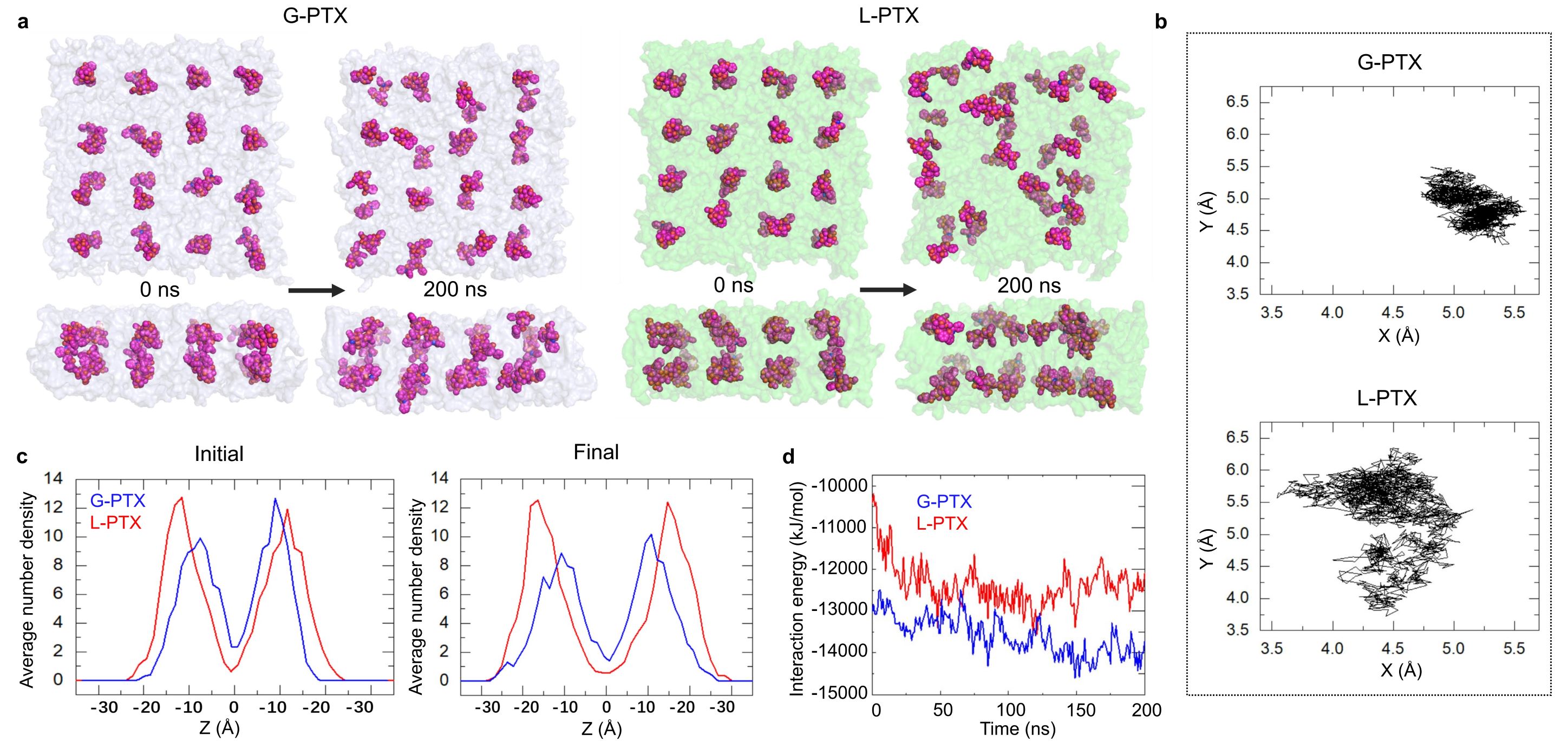


**Fig. S4 The molecular dynamic simulation of G-PTX and control formulation. a** Snapshots of G-PTX and L-PTX membrane model: initial (0 ns) and final (200 ns) views of the G-PTX bilayer and the L-PTX bilayer. The upper row displays the top views and the bottom row displays the side views. Paclitaxel molecules, conventional liposome bilayer, and Rg5-anchored lipid bilayer are colored in purple, green, and light red, respectively. Water molecules are not shown for clarity. **b** Trajectories of a paclitaxel molecule in the G-PTX membrane model and L-PTX membrane model on the X-Y plane. PTX in conventional liposome membrane without hydrogen bond networks underwent random motion in a significantly wider scope of the X-Y plane, compared to the PTX in ginposome membrane. **c** Average number density of PTX in G-PTX (blue) and L-PTX (red) bilayer membrane model at the first and last 10 ns of simulation along the Z-axis, indicating that the PTX was located closer to the center of the membrane in G-PTX than in L-PTX. **d** Interaction energy between the inserted paclitaxel molecule and Rg5-anchored lipid bilayer (G-PTX system, blue), and conventional liposome bilayer (L-PTX, red). It was calculated that the interaction energy between PTX and Rg5-anchored lipid bilayer was approximately 1000 kJ/mol lower than that between PTX and conventional liposome bilayer.

# Figure S5


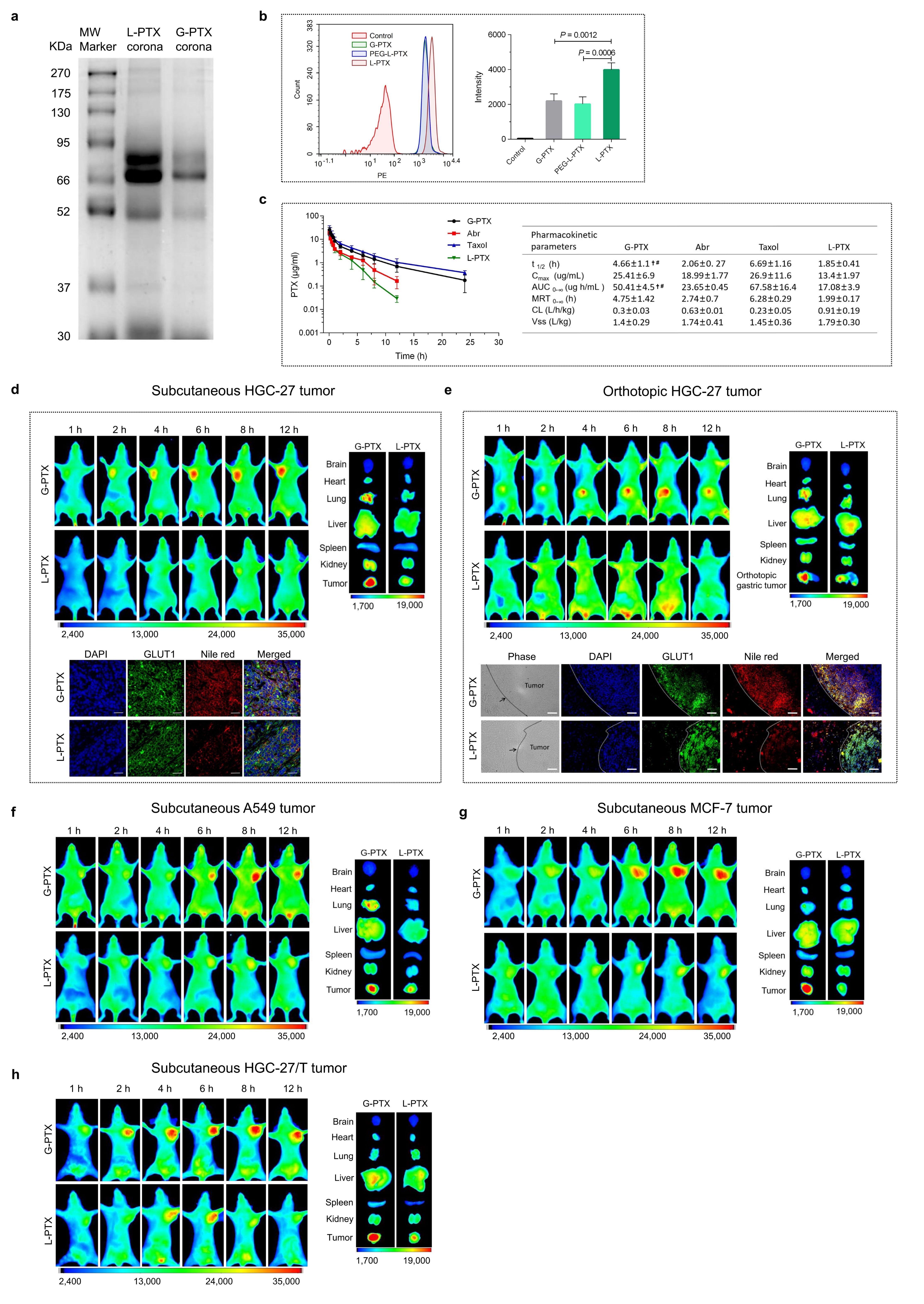


**Fig. S5 The long-circulation property and *in vivo* targeting (by imaging) of G-PTX. a** SDS-PAGE assay of the protein corona absorbed on G-PTX and L-PTX. **b** Cellular uptake studies of G-PTX, L-PTX and PEG-L-PTX in RAW 264.7 cells by flow cytometry (n = 3, one-way ANOVA). Data were shown as mean ± s.d. **c** The plasma concentration-time profiles and pharmacokinetic parameters of G-PTX, Abraxane (Abr), L-PTX and Taxol in rats after i.v. injection at the equivalent dose of 15 mg/kg PTX (n = 3; one-way ANOVA). Data were expressed as mean ± s.d. ^†^*P* < 0.05 versus Abr, ^#^*P* < 0.05 versus L-PTX. Exact *P* values: G-PTX versus Abr, 0.0112 (t_1/2_), 0.0159 (AUC); G-PTX versus L-PTX, 0.0103 (t_1/2_), 0.0048 (AUC). **d**, **e** The targeting ability of IR-783-labeled G-PTX towards subcutaneous (**d**) and orthotopic (**e**) HGC-27 tumors detected by *in vivo* imaging, and relevant *ex vivo* images of the main organs of the tumor-bearing mice at 12 h after intravenous injection. The superior accumulation of G-PTX at the tumor sites in both models and its colocalization with GLUT1 was confirmed by immunofluorescence. Tumor sections were stained with anti-GLUT1, and colocalization of GLUT1 (green) and Nile red-labeled G-PTX (red) was detected. L-PTX was used as control. Scale bar, 25 μm (**d**) and 75 μm (**e**). Arrows (**e**) indicated the area of the HCG-27 tumor. **f**, **g**, **h** The targeted delivery of G-PTX towards A549 (**f**), MCF-7 (**g**), and HGC-27/T (**h**) tumors detected by *in vivo* and *ex vivo* imaging.

# Figure S6


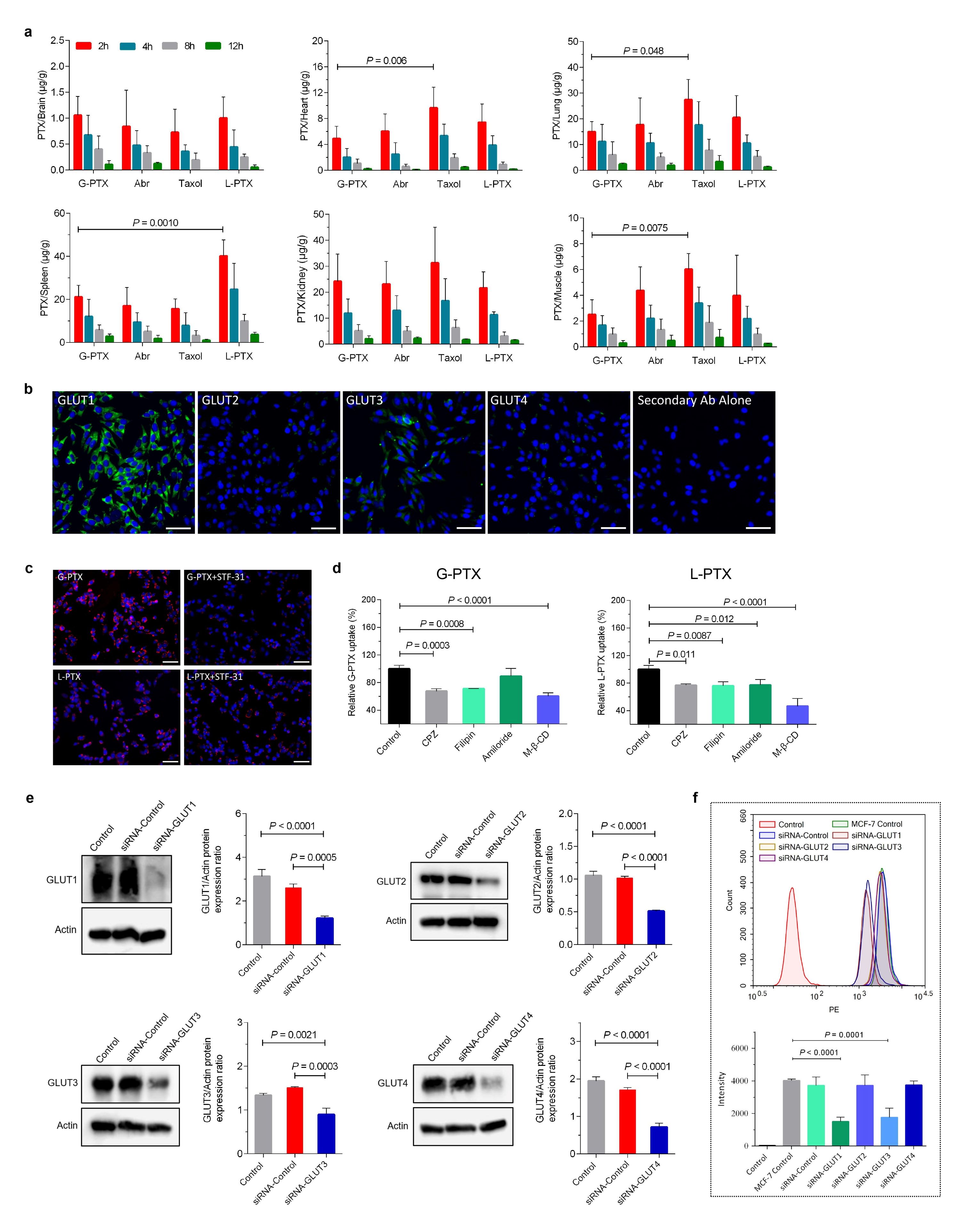


**Fig. S6 The *in vivo* tissue distribution of G-PTX and the active targeting mechanism studies. a** The tissue biodistribution of PTX in HGC-27 tumor-bearing mice treated with G-PTX, Abr, Taxol or L-PTX (n = 3; two-way ANOVA). **b** Immunofluorescence of GLUT1, GLUT2, GLUT3 and GLUT4 in HGC-27 cells. High expression of GLUT1 and low levels of GLUT2, GLUT3 and GLUT4 in HGC-27 cells were observed. Scale bar, 50 μm. **c** The intracellular accumulation of G-PTX in the presence of STF-31 (inhibitor of GLUT1). HGC-27 cells treated with STF-31 showed a significantly reduced G-PTX uptake. Scale bar, 75 μm. **d** The endocytosis mechanism study of G-PTX, by the influence of different uptake inhibitors on the uptake G-PTX and L-PTX by HGC-27 cells (n = 3; one-way ANOVA). **e** Protein levels of GLUT1, GLUT2, GLUT3 and GLUT4 in MCF-7 cells before and after siRNA transfection (n = 3; one-way ANOVA). **f** Cellular uptake by MCF-7 cells with GLUTs 1-4 expression suppressed by siRNA transfection. The cell uptake of G-PTX significantly decreased in MCF-7 cells with suppressed GLUT1 expression (n =3; one-way ANOVA). All data are expressed as mean ± s.d.

# Figure S7


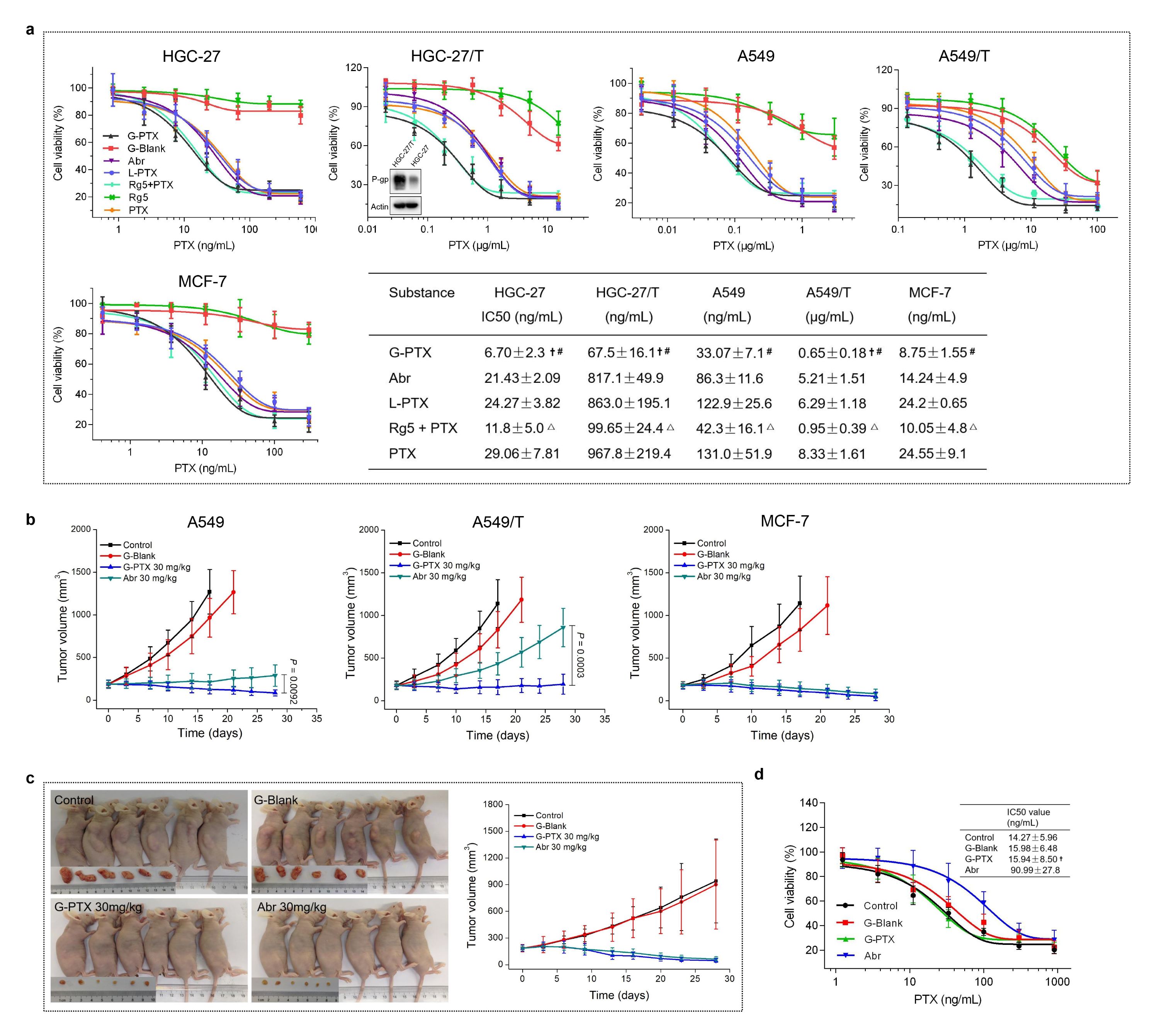


**Fig. S7 The antitumor pharmacodynamics of G-PTX. a** MTT cell viability assay of HGC-27, HGC-27/T, A549, A549/T and MCF-7. Cells were treated with L-PTX, G-Blank, G-PTX or Abr (Abraxane) for 48 h. The mass ratio of Rg5 to PTX was 4:1 (n = 3; one-way ANOVA). ^†^*P* < 0.05 versus Abr, ^#^*P* < 0.05 versus L-PTX, **^△^***P* < 0.05 versus free PTX. Exact *P* values: G-PTX versus Abr, 0.0213 (HGC-27), 0.0003 (HGC-27T), 0.0043 (A549/T); G-PTX versus L-PTX, 0.0070 (HGC-27), 0.0002 (HGC-27T), 0.0168 (A549), 0.0009 (A549T), 0.0276 (MCF-7); Rg5 + PTX versus PTX, 0.0078 (HGC-27), < 0.0001 (HGC-27T), 0.0182 (A549), 0.0001 (A549/T), 0.0395 (MCF-7). **b** Tumor growth curves of A549, A549/T and MCF-7 subcutaneous tumor-bearing mice following the treatment with 120 mg/kg Rg5 content of G-Blank, or 30 mg/kg PTX equivalent concentration of G-PTX and Abr for 4 weeks (n = 6; two-tailed *t*-test). **c** Antitumor activity of G-PTX and Abr in human gastric PDX tumor-bearing mice. Mice bearing gastric PDX tumors received 30 mg/kg PTX equivalent concentration of G-PTX or Abr via tail vein injections for 4 weeks, and the results were shown by tumor volume curve and images of mice with PDX tumor models after treatment, pictures were taken after the mice were sacrificed (n = 6). **d** The sensitivity of HGC-27 tumor after continuous low-dose stimulation with G-Blank, G-PTX or Abr. The cell viability at the presence of free PTX was tested in HGC-27 cells separated from subcutaneous tumor in nude mice which had received G-Blank at a dose of 40 mg/kg Rg5, or G-PTX and Abr at a dose of 10 mg/kg PTX twice per week for 4 weeks (n = 6; one-way ANOVA, ^†^*P* < 0.0001 versus Abr). All data were shown as mean ± s.d.

# Figure S8


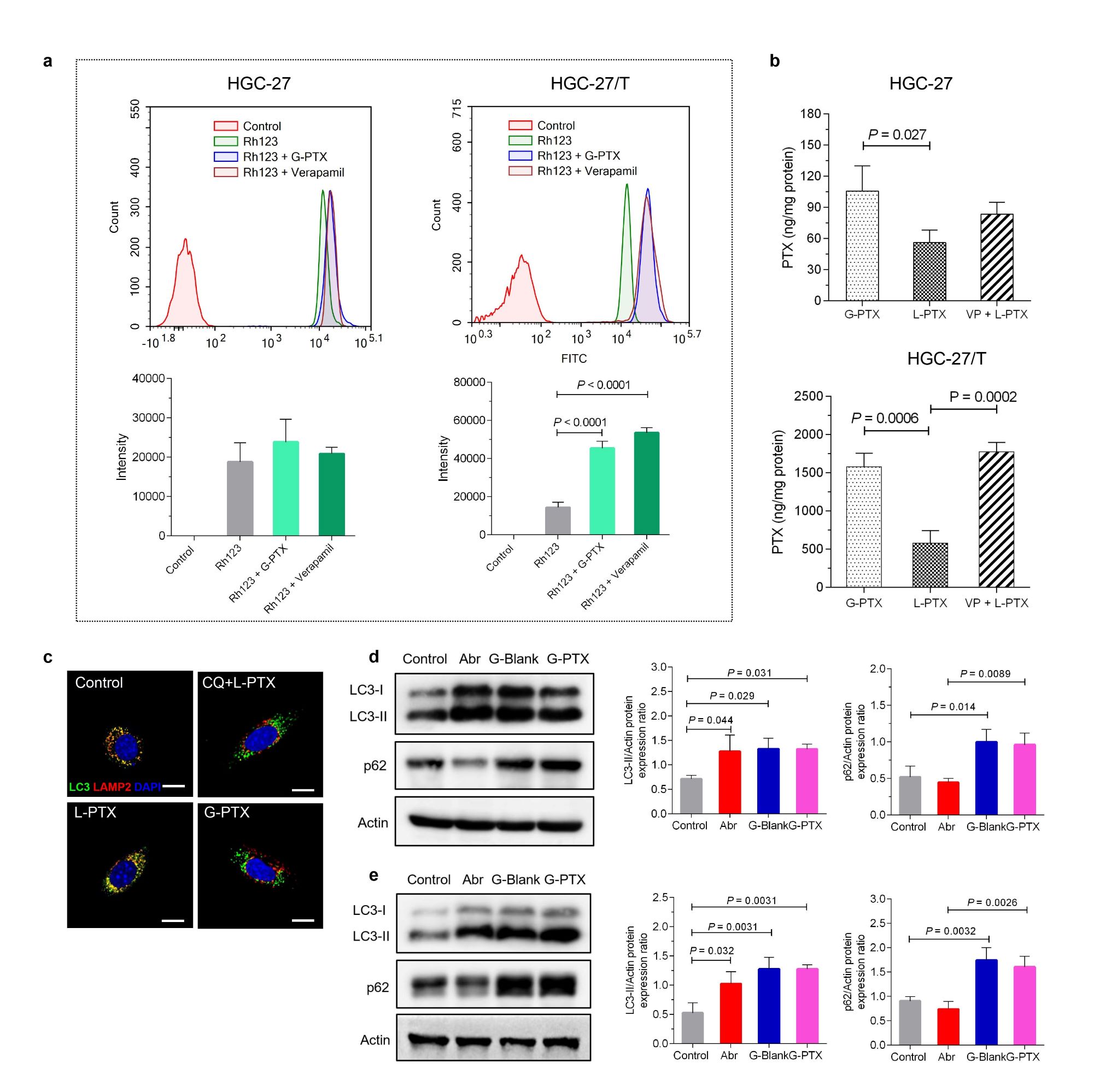


**Fig. S8 The original antitumor mechanism of ginsenoside Rg5 retained by G-PTX: inhibition of P-glycoprotein (P-gp) and the regulation of autophagy in tumor cells. a** Effect of G-PTX on cellular accumulation of Rh123 in HGC-27 cells or HGC-27/T cells. Verapamil, an inhibitor of P-gp, was adopted as control (n = 3; one-way ANOVA). **b** PTX accumulation in HGC-27 cells or HGC-27/T cells after incubation with G-PTX, L-PTX and L-PTX + Verapamil (VP) at 37 ℃ for 2 h (n = 3; one-way ANOVA). **c** Immunofluorescence analysis of autophagosome-lysosome fusion in HGC-27/T cells after the treatment of G-PTX or L-PTX. Autophagosomes were labeled by antibody against LC3, and lysosomes were labeled by antibody against LAMP2. Chloroquine (CQ) served as a positive control. Scale bar, 10 μm. **d** Autophagy protein immunoblotting of HGC-27/T tumor after systemic administration of G-PTX, Abr (30 mg PTX/kg) or G-Blank (120 mg Rg5/kg) once per week for 4 weeks (n = 3; one-way ANOVA). **e** Western blot analysis of autophagy protein expression in the HGC-27 subcutaneous tumor treated twice weekly with G-PTX or Abr at a dose of 10 mg/kg PTX for 4 weeks (n = 3; one-way ANOVA).

# Figure S9


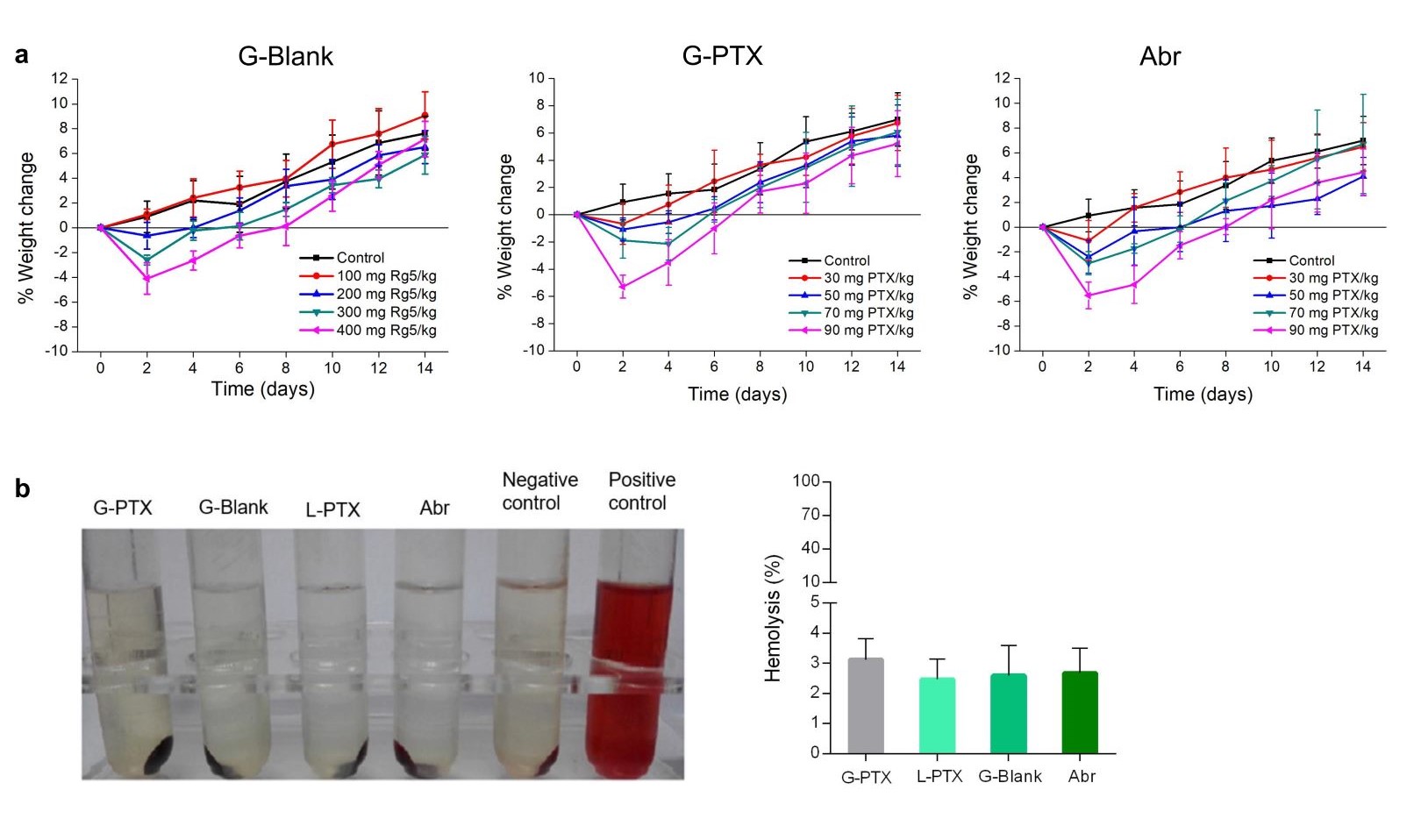


**Fig. S9 The safety evaluation of G-PTX: maximum tolerable dose (MTD) and hemolysis. a** Body weight of mice expressed as a percentage of their starting weight following treatment with G-Blank, G-PTX or Abraxane (Abr). No significant weight loss was observed in these groups (n = 6). **b** Hemolysis test of different formulations at PTX concentration of 0.25 mg/mL (G-PTX, L-PTX and Abr) and Rg5 concentration of 1 mg/mL (G-Blank). n = 3. All data were expressed as mean ± s.d.

# Figure S10

**
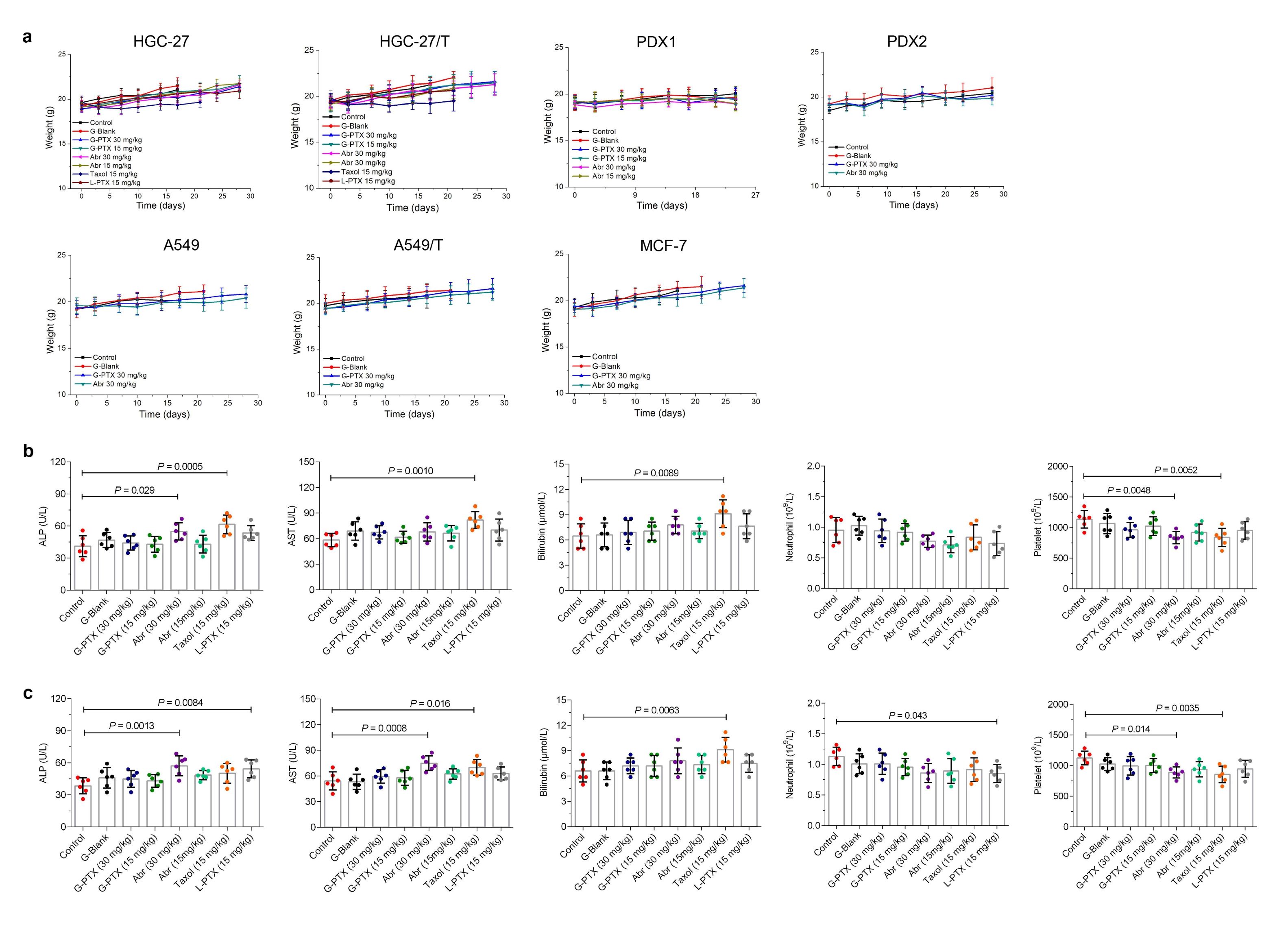
**

**Fig. S10 The safety evaluation of G-PTX: body weight, haemocytes counts and blood biochemical indicators. a** Changes in the body weight of 7 different tumor-bearing nude mice models treated with various drug-loaded formulations. Under high-dose continuous administration of 30 mg/kg PTX, the body weight of the G-PTX treatment group did not change significantly during the entire treatment course (n = 6). **b**, **c** Haemocytes counts and levels of blood biochemical indicators following the treatment of G-Blank, G-PTX, Abr, Taxol or L-PTX in HGC-27 (**b**) and HGC-27/T (**c**) tumor-bearing mice. No significant changes in platelets and neutrophils in mice treated with G-PTX were observed. Compared with the control group, the blood biochemical indicators of the high-dose Abr group (30 mg/kg), Taxol group and L-PTX group increased to varying degrees, while the high-dose and low-dose of G-PTX groups showed no significant changes in blood biochemical indicators (n = 6; one-way ANOVA). All data were shown as mean ± s.d.

References

1. Oh, S. J. et al. Sorafenib decreases proliferation and induces apoptosis of prostate cancer cells by inhibition of the androgen receptor and Akt signaling pathways. *Endocr. Relat. Cancer* **19**, 305–319 (2012).
2. Ravar, F. et al. Hyaluronic acid-coated liposomes for targeted delivery of paclitaxel, in-vitro characterization and in-vivo evaluation. *J. Controlled Release* **229**, 10–22 (2016).
3. Salomon-Ferrer, R., Case, D. A. & Walker, R. C. An overview of the Amber biomolecular simulation package. *WIREs Comput. Mol. Sci.* **3**, 198–210 (2013).
4. Case, D. A. et al. The Amber biomolecular simulation programs. *J. Comput. Chem*. **26**, 1668–1688 (2005).
5. Wu, E. L. et al. CHARMM-GUI Membrane Builder toward realistic biological membrane simulations. *J. Comput. Chem.* **35**, 1997–2004 (2014).
6. Jo, S., Lim, J. B., Klauda, J. B. & Im, W. CHARMM-GUI Membrane Builder for mixed bilayers and its application to yeast membranes. *Biophys. J.* **97**, 50–58 (2009).
7. Lee, J. et al. CHARMM-GUI input generator for NAMD, GROMACS, AMBER, OpenMM, and CHARMM/OpenMM simulations using the CHARMM36 additive force field. *J. Chem. Theory Comput.* **12**, 405–413 (2016).
8. Abraham, M. J. et al. GROMACS: High performance molecular simulations through multi-level parallelism from laptops to supercomputers. *SoftwareX* **1–2**, 19–25 (2015).
9. Pronk, S. et al. GROMACS 4.5: a highthroughput and highly parallel open source molecular simulation toolkit. *Bioinformatics* **29**, 845–854 (2013).
10. Waterhouse, A. et al. SWISS-MODEL: homology modelling of protein structures and complexes. *Nucleic Acids Res.* **46**, W296–W303 (2018).
11. Guex, N., Peitsch, M. C. & Schwede, T. Automated comparative protein structure modeling with SWISS-MODEL and Swiss-PdbViewer: a historical perspective. *Electrophoresis* **30**, S162–173 (2009).
12. Benkert, P., Biasini, M. & Schwede, T. Toward the estimation of the absolute quality of individual protein structure models. *Bioinformatics* **27**, 343–350 (2011).
13. Friesner, R. A. et al. Glide: a new approach for rapid, accurate docking and scoring. 1. Method and assessment of docking accuracy. *J. Med. Chem.* **47**, 1739–1749 (2004).
14. Halgren, T. A. et al. Glide: a new approach for rapid, accurate docking and scoring. 2. Enrichment factors in database screening. *J. Med. Chem.* **47**, 1750–1759 (2004).
15. Friesner, R. A. et al. Extra precision glide: docking and scoring incorporating a model of hydrophobic enclosure for protein-ligand complexes. *J. Med. Chem.* **49**, 6177–6196 (2006).
16. Kure, T. & Sakai, H. Transmembrane difference in colloid osmotic pressure affects the lipid membrane fluidity of liposomes encapsulating a concentrated protein solution. *Langmuir* **33**, 1533–1540 (2017).
17. Shimizu, K. et al. Temperature-dependent transfer of amphotericin B from the liposomal membrane of AmBisome to fungal cell membrane. *J. Controlled Release* **141**, 208–215 (2010).
18. Lin, Z. et al. Novel thermo-sensitive hydrogel system with paclitaxel nanocrystals: High drug-loading, sustained drug release and extended local retention guaranteeing better efficacy and lower toxicity. [*J. Controlled Release*](https://www.ncbi.nlm.nih.gov/pubmed/?term=Novel+thermo-sensitive+hydrogel+system+with+paclitaxel+nanocrystals:High+drug-loading,+sustained+drug+release+and+extended+local+retention+guaranteeing+better+efficacy+and+lower+toxicity) **174**, 161–170 (2014).
19. Rigaud, J. L. & [Lévy](https://e.glgoo.top/citations?user=xmOaZ8EAAAAJ&hl=zh-CN&oi=sra), D. Reconstitution of membrane proteins into liposomes. *Methods Enzymol*. **372**, 65–86 (2003).
20. Renauld, S. et al. Functional reconstitution of cell-free synthetized purified Kv channels. *BBA-Biomembranes* **1859**, 2373–2380 (2017).
21. [Corbo, C](https://www.ncbi.nlm.nih.gov/pubmed/?term=Corbo%20C%5bAuthor%5d&cauthor=true&cauthor_uid=27445473). et al. effects of the protein corona on liposome-liposome and liposome-cell interactions. [*Int. J. Nanomedicine*](https://www.ncbi.nlm.nih.gov/pubmed/?term=effects+of+the+protein+corona+on+liposome%E2%80%93+liposome+and+liposome%E2%80%93cell+interactions) **11**, 3049–3063 (2016).
22. Li, H. et al. Histology and antitumor activity study of PTX-loaded micelle, a fluorescent drug delivery system prepared by PEG-TPP. *Chin. Chem. Lett.* **30**, 1083–1088 (2019).
23. Furukawa, T. et al. Nude mouse metastatic models of human stomach cancer constructed using orthotopic implantation of histologically intact tissue. *Cancer Res.* **53**, 1204–1208 (1993).
24. [Yang, X](https://www.ncbi.nlm.nih.gov/pubmed/?term=Yang%20X%5bAuthor%5d&cauthor=true&cauthor_uid=25904021). et al. Nsc23925 prevents the development of paclitaxel resistance by inhibiting the introduction of P-glycoprotein and enhancing apoptosis. [*Int. J. Cancer*](https://www.ncbi.nlm.nih.gov/pubmed/?term=Nsc23925+prevents+the+development+of+paclitaxel+resistance+by+inhibiting+the+introduction+of+P-glycoprotein+and+enhancing+apoptosis) **137**, 2029–2039 (2015).
25. Zhou, H. et al. [IGF1 receptor targeted theranostic nanoparticles for targeted and image-guided therapy of pancreatic cancer](https://pubs.acs.org/doi/abs/10.1021/acsnano.5b01288). [*ACS Nano* **9**, 7976–7991 (2015).](https://www.ncbi.nlm.nih.gov/entrez/eutils/elink.fcgi?dbfrom=pubmed&retmode=ref&cmd=prlinks&id=26242412)
26. Song, E.-K. et al. Potent antitumor activity of cabozantinib, a c-MET and VEGFR2 Inhibitor, in a colorectal cancer patient-derived tumor explant model. *Int. J. Cancer* **136**, 1967–1975 (2015).
27. [Luo, T](https://www.ncbi.nlm.nih.gov/pubmed/?term=Luo%20T%5bAuthor%5d&cauthor=true&cauthor_uid=27515664). et al. PEGylation of paclitaxel largely improves its safety and anti-tumor efficacy following pulmonary delivery in a mouse model of lung carcinoma. [*J. Controlled Release*](https://www.ncbi.nlm.nih.gov/pubmed/?term=PEGylation+of+paclitaxel+largely+improves+its+safety+and+anti-tumor+efficacy+following+pulmonary+delivery+in+a+mouse+model+of+lung+carcinoma) **239**, 62–71 (2016).
